# A primary cell wall cellulose-dependent defense mechanism against vascular pathogens revealed by time-resolved dual transcriptomics

**DOI:** 10.1186/s12915-021-01100-6

**Published:** 2021-08-17

**Authors:** Alexandra Menna, Susanne Dora, Gloria Sancho-Andrés, Anurag Kashyap, Mukesh Kumar Meena, Kamil Sklodowski, Debora Gasperini, Nuria S. Coll, Clara Sánchez-Rodríguez

**Affiliations:** 1 Department of Biology, ETH Zürich, 8092 Zürich, Switzerland; 2 Centre for Research in Agricultural Genomics (CRAG), CSIC-IRTA-UAB-UB, 08193 Barcelona, Spain; 3 Department of Molecular Signal Processing, Leibniz Institute of Plant Biochemistry, 06120 Halle (Saale), Germany

**Keywords:** Arabidopsis, *Fusarium oxysporum*, *Ralstonia solanacearum*, plant-pathogen interaction, dual-time course transcriptomics, cellulose, ethylene, defense response

## Abstract

**Background:**

Cell walls (CWs) are protein-rich polysaccharide matrices essential for plant growth and environmental acclimation. The CW constitutes the first physical barrier as well as a primary source of nutrients for microbes interacting with plants, such as the vascular pathogen *Fusarium oxysporum* (Fo). Fo colonizes roots, advancing through the plant primary CWs towards the vasculature, where it grows causing devastation in many crops. The pathogenicity of Fo and other vascular microbes relies on their capacity to reach and colonize the xylem. However, little is known about the root-microbe interaction before the pathogen reaches the vasculature and the role of the plant CW during this process.

**Results:**

Using the pathosystem Arabidopsis-Fo5176, we show dynamic transcriptional changes in both fungus and root during their interaction. One of the earliest plant responses to Fo5176 was the downregulation of primary CW synthesis genes. We observed enhanced resistance to Fo5176 in Arabidopsis mutants impaired in primary CW cellulose synthesis. We confirmed that Arabidopsis roots deposit lignin in response to Fo5176 infection, but we show that lignin-deficient mutants were as susceptible as wildtype plants to Fo5176. Genetic impairment of jasmonic acid biosynthesis and signaling did not alter Arabidopsis response to Fo5176, whereas impairment of ethylene signaling did increase vasculature colonization by Fo5176. Abolishing ethylene signaling attenuated the observed resistance while maintaining the dwarfism observed in primary CW cellulose-deficient mutants.

**Conclusions:**

Our study provides significant insights on the dynamic root-vascular pathogen interaction at the transcriptome level and the vital role of primary CW cellulose during defense response to these pathogens. These findings represent an essential resource for the generation of plant resistance to Fo that can be transferred to other vascular pathosystems.

**Supplementary Information:**

The online version contains supplementary material available at 10.1186/s12915-021-01100-6.

## Background

All living organisms must adapt to their environment to survive and reproduce in their habitats. This is particularly challenging for sessile organisms like plants, which rely on remarkable plasticity to adjust to different and simultaneous external cues. In addition, plant cells are immobile, so each of them is fully equipped with sophisticated molecular artillery to perceive and respond to incoming stresses [1]. The plant cell wall (CW), a rigid yet dynamic polysaccharide-protein matrix, is an essential player in plant responses to external stimuli. The CW acts as the first physical barrier to outside invaders or stresses and as a source of signals to trigger downstream responses upon perception of incoming danger [2]. Moreover, plant acclimation to the environment relies on accurate developmental changes that depend on the precise remodeling of the CWs [1, 3]. Therefore, plant CW alteration directly influences growth and stress response pathways. This is especially relevant during plant response to microbes who mainly live in the apoplast, like the root vascular pathogen *Fusarium oxysporum* (Fo).

Fo is a soil-borne root-infecting hemibiotrophic fungal pathogen, responsible for the devastation of many economically important crop species such as banana, tomato, cotton, and cabbage [4]. Fo attaches to the outer epidermal root cell layer to find wounds or weak points to penetrate these outer cell layers [4, 5]. Hyphae then advance towards the xylem, where fungal proliferation blocks water and nutrient uptake, causing wilting and eventually plant death. Because of these dramatic symptoms, most studies characterize Fo infections in aerial plant tissues, while the essential root-colonization stage remains poorly understood due to the difficulty in accessing this plant organ. Moreover, this infection phase preceding Fo penetration into the xylem is described as asymptomatic based on the absence of aerial infection symptoms, despite the fact that roots already begin to exhibit evidence of response to stress at this stage [4]. Various phytohormones, including salicylic acid (SA), jasmonic acid (JA), and ethylene (ET), among others, have been implicated in plant response to various Fo strains [6–8]*.* Fo is genetically diverse, with different strains grouped based on narrow host ranges [4, 9]. Despite our knowledge of Fo host specificity, details of the specific infection strategies and plant defense mechanisms are still unclear for many Fo-plant pathosystems. Fo5176 infects the model plant *Arabidopsis thaliana*, constituting an ideal pathosystem to study root colonization of vascular pathogens [10, 11]. Some studies have used this pathosystem to provide relevant information regarding root-mediated and tissue-specific defense responses to Fo. Novel aspects of plant defense to Fo5176 have been identified, including reactive oxygen species (ROS) production [12], as well as enhanced auxin and abscisic acid signaling [13]. The conclusions from these studies reflect plant responses during the biotrophic colonization phase (1 and 2 days after treatment (dpt) with spores in [12, 13]), or when Fo is potentially transitioning from a biotrophic to a necrotrophic lifestyle (6 dpt in [13]). Therefore, a deeper understanding of Fo infection progression inside the root at a higher temporal and spatial resolution is necessary. In its path towards the root vasculature, Fo passes through plant CWs. Therefore, as other microbes, Fo modifies and degrades the plant CW polysaccharides during host colonization [14]. To date, many aspects of this essential plant CW degradation and modification processes remain largely unknown.

Plant root cells, with the exception of the vascular, periderm, and the differentiated endodermis cells, have only primary CWs. Cellulose is one of the most abundant polymers in primary CWs and provides the majority of the load-bearing strength of the plant CW [15, 16]. Cellulose is synthesized at the plasma membrane by cellulose-synthase (CESA) complexes, which extrude glucan chains into the apoplast guided by cortical microtubules [17, 18]. Mutations in the Arabidopsis primary CW CESA subunits, like *prc1-1* impaired in CESA6, lead to significant reduction in cellulose content that results in abnormal cell elongation and dwarfism [19, 20]. Similar phenotypes are observed in Arabidopsis plants compromised in the activity of the apoplastic chitinase-like 1 (CTL1), the glycophosphatidyl-inositol (GPI)-linked COBRA (COB), and the PM-bound endo-1,4,-β-glucanase KORRIGAN1 (KOR1), also required for primary CW-cellulose synthesis [19, 21–23]. The biological response to genetic or chemically induced primary CW-cellulose deficiency includes ectopic lignification, upregulation of stress-related genes, and accumulation of the phytohormones JA, ET and SA [24]. These transcriptional and cellular changes have been associated with increased resistance to pathogens of primary CW cellulose-deficient mutants, as lignin deposition restricts pathogen infection [25] and JA, ET, and SA are well-known players in plant defense [7, 26, 27]. However, evidence connecting primary CW cellulose mutants with biotic stress response has only been described for the *cesa3/cev1* mutant, impaired in the primary CW CESA subunit CESA3 [28, 29]. The constitutive activation of JA/ET signaling pathways in the *cesa3/cev1* mutant, typically associated with primary CW cellulose-deficiency, contributed to its enhanced resistance to three leaf-biotrophic pathogens [30]. However, the precise roles of primary CW perturbations or hormonal fluxes in pathogen defense remain to be fully clarified. While many studies characterized defense mechanisms activated in response to leaf-infecting pathogens, the role of hormones during root-infecting pathogen invasion is largely under-studied [31]. Furthermore, the complex interaction between Fo and its host suggests a precisely fine-tuned spatio-temporal communication at the root primary CWs, but this dialogue remains largely unknown.

To investigate the Fo-root interaction prior to xylem colonization, we first classified different colonization stages by confocal microscopy, and then performed a time course dual transcriptome study. Our data uncovered a fast and relevant role of primary CW cellulose modulation in plant response to Fo. We observed that primary CW cellulose-deficiencies directly impact Fo5176 infection by facilitating fungal colonization. By further elucidating ways through which primary CW-cellulose mutants mitigate vascular pathogen invasion, we shed light on the inextricably linked connections between primary CWs, phytohormone-signaling, and defense response activation.

## Results

### Arabidopsis root and Fo5176 transcriptomics reveal temporal acclimations during their interaction

**Fig. 1:**
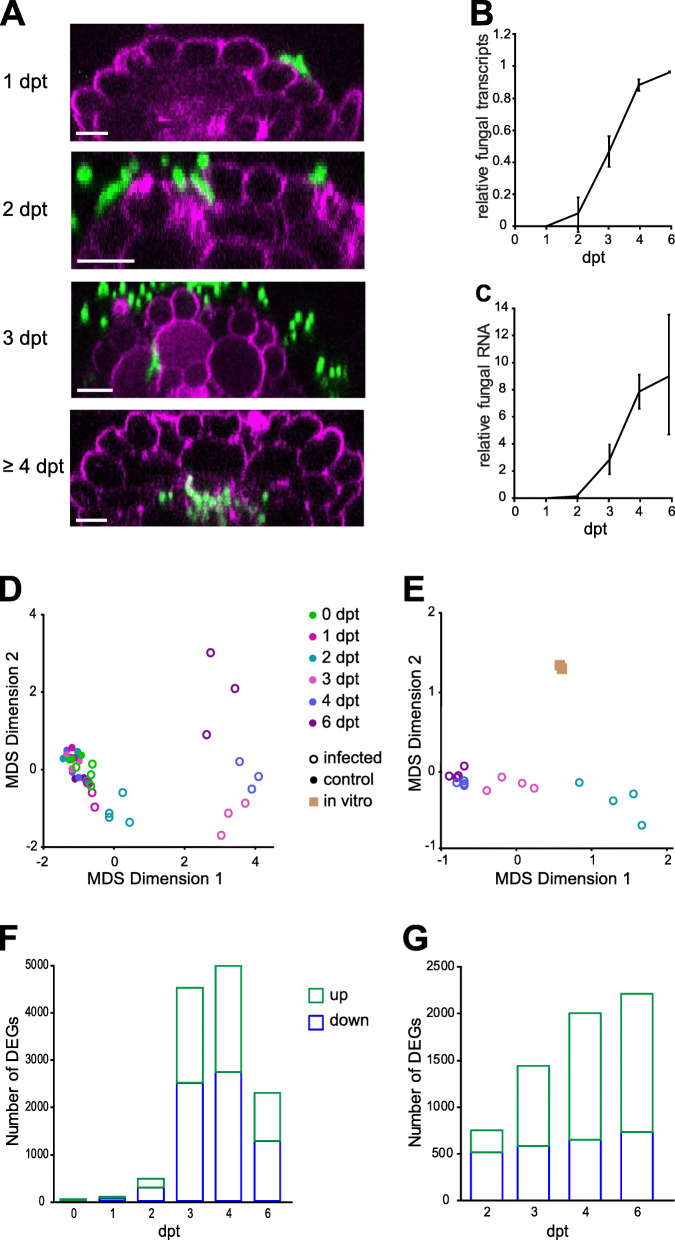
Fo5176 infection of Arabidopsis roots leads to temporal dynamic changes in the plant and the fungal transcriptomes. **A** Microscopic analysis of root infection by Fo5176 over 4 days after microconidia treatment (dpt). Left panels: Representative confocal images of Fo5176 (green) colonizing the different cell layers of Arabidopsis roots (magenta). Scale bars 20 μm. Each image represents a minimum of 9 hydroponically infected and 4 plate-infected roots/dpt. **B** Percentage of transcripts based on the RNA-seq analysis mapped to the fungal reference genome at all investigated time points using a splice aware sequence aligner (STAR). Values represent the mean ± standard error of four biological replicates. **C** Fo5176 biomass determination over time based on RNA and determined by qRT-PCR using a fungus-specific primer (Fo5176 β-Tub) relative to an Arabidopsis reference gene (At GAPDH). Values represent the mean ± standard error of four biological replicates. **D**, **E** Multidimensional scaling analysis (MDS) of transcriptional profiles of Arabidopsis (**D**) and Fo5176 (**E**). **F**, **G** Number of differentially expressed genes (DEGs) upregulated (green) or downregulated (blue) at each root colonization stage (0-6 dpt and 2-6 dpt, respectively) in Arabidopsis (**F**) and Fo5176 (**G**)

To study the Fo5176 infection progression at the microscopic level, we imaged the roots of hydroponically and plate-grown Arabidopsis plants exposed to the fluorescently labeled strain Fo5176 pGPD::GFP [11] over a period of 6 days (Figure 1A). Microconidia germination and attachment to the root were observed at 1 dpt. Fungal hyphae entered the roots at 2 dpt, mainly at emerging lateral roots, colonizing the apoplastic space of the epidermis layer. At 3 dpt, the hyphae were visible in the cortex both inter- and intracellularly. At 4 dpt, the first fungal vascular penetrations were observed in some plants and all roots had at least one vascular penetration event at 6 dpt.

We then explored the temporal transcriptional changes in both Fo5176 and Arabidopsis roots at the identified colonization stages (Figure 1A). For each time point, mock-treated roots were included and four biological replicates were generated. All samples were collected at the middle of the day to reduce the influence of the circadian clock in the results. As additional references, we included Fo5176-treated roots for only 30 min, when no microconidia germination was observed (0dpt), and Fo5176 exponentially grown in vitro (“in vitro”). By Illumina sequencing of 3’mRNA libraries, we obtained more than 234 billion reads from all samples, which were mapped to the Arabidopsis TAIR10 gene models [32] and the Fo5176 genome [10] (Additional file 1: Table S1). These data have been deposited in the NCBI Gene Expression Omnibus [33] and are accessible through the GEO series accession number GSE168919. For the early time points of 0 dpt and 1 dpt, the number of reads mapping to the fungal genome was too low to include them in the analysis (< 4300 and < 10,800, respectively; Additional file 1: Table S1). The fungal reads steadily increased from 2 dpt on, reaching 50% at 3 dpt, and represented the vast majority of reads at 4 and 6dpt (Figure 1B). Despite the low % of plant reads at the two last time points, their number is high enough to consider them for further analysis (~ 43,000 and > 20,000, respectively; Additional file 1: Table S1). The number of fungal mapped reads correlated with the increased fungal biomass quantified during root colonization (Figures 1B and C). Only those genes represented with more than 3 counts per million (CPM) across all samples and conditions (almost 58% of the Arabidopsis genes and 46% of Fo5176; Additional file 1: Table S1) were considered to be actively expressed and included in further analysis.

To determine overall changes in the host and the pathogen transcriptomes over time, a multi-dimensional scaling (MDS) analysis was performed. Following this approach, 5 samples from the plant mapped reads that did not cluster with the rest of the samples from the same time point were identified: 0 dpt mock, 1 dpt Fo, 3 dpt Fo, 4 dpt Fo, and 6 dpt Fo (Additional file 2: Figure S1). After the removal of these 5 samples, we continued with the analysis of at least 3 independent biological replicates for each investigated time point under control and infection conditions (Figure 1D). Fo5176 mapped reads from 0 and 1 dpt were excluded from the analysis due to the insufficient amount of reads obtained for those time points. In both organisms, the infected samples formed clusters distinct from control-treated samples starting at 2 dpt, and the largest difference between infected clusters was observed between 2 dpt and 3 dpt (Figure 1D and E). These results suggest different behavior of plant and pathogen transcript profiles when the fungus reaches the root cortex (2 to 3 dpt; Figure 1A). Moreover, Fo5176 transcriptomes at 4 and 6 dpt were very similar, coinciding with the fungal entrance in the vasculature. This indicates that the fungal gene expression pattern changes along with continued growth towards the vasculature and accumulation of fungal biomass in the root and seems to be tissue-dependent. We continued the transcriptomic study by comparing gene expression at all time points, i.e., 0–6 dpt for Arabidopsis and 2–6 dpt for Fo5176 (Figures 1F and G). These analyses revealed a total of 7053 plant genes and 2902 fungal genes being differentially expressed (DEGs) at least at one time point (adj. *p* value < 0.05, log_2_FC > |1|; Figures 1F and G, Additional file 3 and 4: Tables S2 and S3, respectively). The host transcriptome was enriched in downregulated DEGs, while we detected more DEGs to be upregulated than downregulated in Fo5176 (Figure 1F and G). To validate the expression data, six genes were randomly picked from each organism and their expression levels were evaluated by qRT-PCR from de novo generated RNA samples (Additional file 5: Figure S2A and S2B). We observed a strong linear correlation (*r* = 0.79 for Arabidopsis and *r* = 0.97 for Fo5176) between the qRT-PCR and RNA-seq data (Additional file 5: Figure S2C and S2D).

DEGs were further analyzed for their expression pattern over time by fuzzy c-means clustering [34]. This tolerant clustering approach was selected to enable the sorting of genes to centroids depending on the similarity of their expression profile over time and their membership to the cluster. Based on the membership threshold, a gene can be present in more than one cluster. The fungal DEGs grouped in 5 expression clusters showing different temporal gene expression patterns (Additional file 4: Table S3 and Additional file 6: Figure S3A): increase (clusters 1 and 2), increase-decrease (clusters 3 and 4), or decrease (cluster 5). Five fungal DEGs were excluded from clustering, as they do not show any expression in planta (g14801, g5058, g8821, g8822, g8995). We observed that the gene expression profiles of different clusters peak at different days, suggesting that genes contained in these clusters serve a function during that specific stage of the infection. We aimed to further identify the transcriptomic profile of fungal metabolism involved in plant cell wall modification. Due to limited Gene Ontology (GO)-annotations available for Fo5176, we focused our analyses on those containing a Carbohydrate Active enZYme (CAZY) domain. We further separated glycosyl hydrolases that might act directly on cell wall moieties from other cell wall related functions (Additional file 4: Table S3). The majority of the genes encoding for cell wall related genes clustered together in cluster 3 (Additional file 6: Figure S3B), whose expression progressively increases until 4dpt, when the fungus reaches the vasculature.

**Fig. 2:**
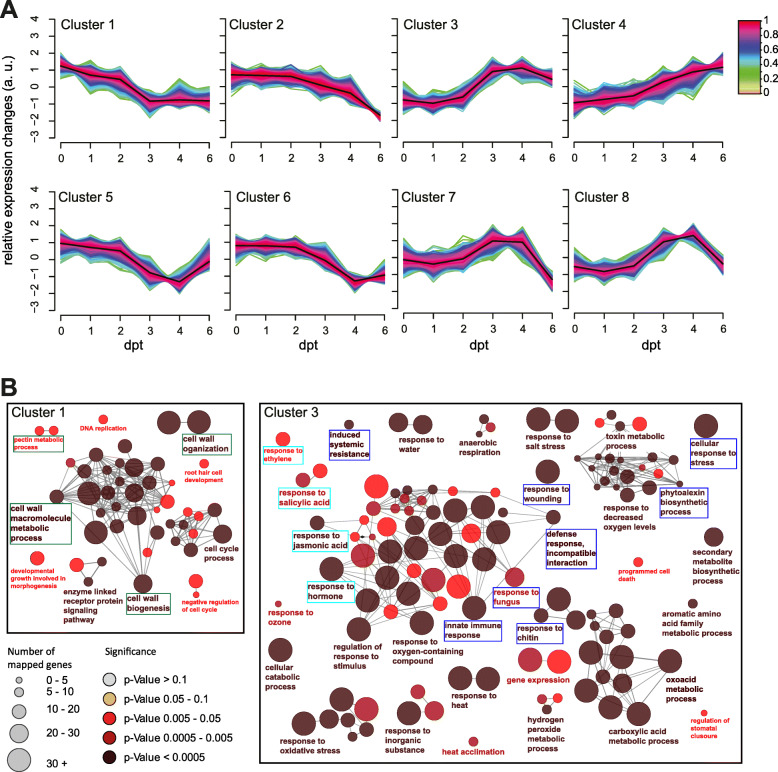
Temporal dynamics of Arabidopsis DEGs during infection reveal a significant alteration of cell wall biology and hormonal process in response to Fo5176. **A** Clusters of Arabidopsis coexpressed DEGs during infection using fuzzy c-means clustering. **B** Biological Processes Enriched in Selected Clusters from (**A**) using GO enrichment analysis. Enriched GO-terms are depicted as circles, lines connecting the circles show relation between GO-terms. In each group of GO-terms, the most significant category is labeled with the GO-term description. The genes downregulated over time during Fo5176 root colonization (coexpressed in cluster 3 in (**A**)) are enriched in plant cell wall biological processes (left panel, green). Cluster 7 (right panel) represents general defense responses (dark blue) and responses to hormonal signaling (light blue)

Fuzzy clustering of the gene expression profiles for the Arabidopsis DEGs identified 8 expression clusters showing different temporal gene expression patterns (Fig. 2A): decrease (clusters 1 and 2), increase (clusters 3 and 4), decrease-increase (clusters 5 and 6), or increase-decrease (clusters 7 and 8). To obtain a picture of the biological processes associated with root response to Fo5176, each cluster was subjected to a GO term enrichment analysis (Additional file 3: Table S2) [35]. This analysis revealed that downregulated genes are enriched in biological processes related to cell wall synthesis and remodeling: plant-type cell wall biogenesis (GO:0009832), plant-type cell wall organization (GO:0009664), plant-type secondary cell wall biogenesis (GO:0009834), and cell wall polysaccharide metabolic process (GO:0010383). Among others, we detected the downregulation of seven out of the ten cellulose synthase (*CESAs*) transcripts, *CESA1*, *CESA2*, *CESA3*, *CESA4*, *CESA5*, *CESA7*, and *CESA8* [36], other cellulose synthesis genes, *CTL1* and *CTL2* *[*21*]*, and more than 20 arabinogalactan proteins (AGPs) [37]. Cluster 1 was particularly enriched in these GO categories (Figure 2B and Additional file 4: Table S3). Conversely, upregulated genes from 3 dpt showed overall enrichment in biological processes associated with defense (Additional file 3: Table S2): plant-type hypersensitive response (GO:0009626), camalexin metabolic process (GO:0052317), innate immune response (GO:0045087), defense response to fungus (GO:0050832), JA, ET, and SA responses (GO:0009753, GO:0009723, GO:0009751), and JA-mediated signaling (GO:2000022, GO:0009867). Among them, we found chitinase family proteins associated with the CAZY-family GH19 (Additional file 3: Table S2), potentially needed for defense against fungal pathogens; several plant defensins [38]; genes reported to be involved in plant immune responses: *WRKY33* [39], *PR4* [40], *PEPR1* [41]; peroxidases (*PRX33*, *PRX34* [42];); and hormone-related genes like *JAZs* repressors and *ERFs* transcription factors [43–45]. Many of these genes clustered together in cluster 3 (Figure 2B, Additional file 3: Table S2). Taken together, these results suggest that infected Arabidopsis roots undergo major transcriptional reprogramming leading to overall repression of growth followed by activation of stress and defense responses.

### Downregulation of primary cell wall cellulose synthesis results in enhanced Fo5176 resistance

**Fig. 3:**
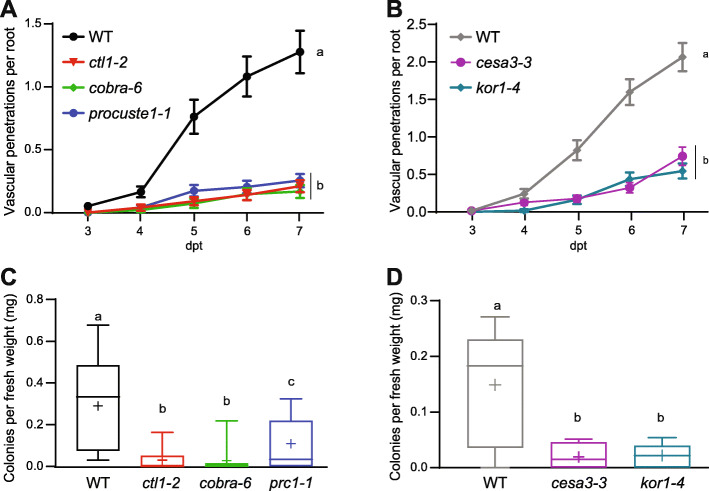
Primary cell wall cellulose-deficient mutants exhibit enhanced resistance to Fo5176. **A**, **B** Root vascular penetration of WT (Col-0 in (**A**) and Col-0 JGP in (**B**)) and primary cellulose deficient mutants at various days post-treatment (dpt) with Fo5176 pSIX1::GFP microconidia. Values represent the mean ± standard error of at least 3 independent experiments, each one containing at least 12 seedlings. Statistical significance calculated via repeated measures two-way ANOVA with Tukey post-hoc test (*p* value ≤ 0.05 (genotype), *p* value ≤ 0.05 (time), *p* value ≤ 0.05 (genotype x time)). Significant differences at the last time point shown (7 dpt) are indicated on the graph using letters; statistics of remaining time points summarized in Additional file 9: Table S4 A-F. **C**, **D** Quantification of Fo5176 pSIX1::GFP colonies after surface sterilization of infected roots at 7dpt. Box plots: centerlines show the medians; box limits indicate the 25th and 75th percentiles; whiskers extend to the minimum and maximum. N ≥ 3 independent experiments, each one containing at least 6 roots. Statistical significance was calculated via one-way ANOVA with a Tukey post-hoc test (*p* value ≤ 0.05)

We observed significant downregulation of genes encoding for proteins involved in primary cell wall cellulose synthesis from 3 dpt on: *CESAs*, *CTL1*, *COBRA*, and *KOR*1 (Additional file 3: Table S2). Plant roots are predominantly surrounded by cellulose-rich primary CWs. Therefore, we sought to determine the potential outcome of primary CW-cellulose synthesis reduction during plant response to Fo5176. We hence characterized the infection phenotypes of the corresponding previously described primary CW cellulose-deficient mutants *ctl1-2*, *cobra-6*, *procuste1-1*, and *kor1-4* *[*20*–*22, 27*]* (Additional file 7: Figure S4) and a new allele of *cesa3* and *cesa3-3* (Additional file 8: Figure S5). In comparison to characterized mutant alleles in CESA3 such as *cev1* and *ixr1*, the novel *cesa3-3* allele has a milder root growth phenotype that is more amenable to Fo infection assays, while maintaining the typical root cell swelling and increased lignin deposition of primary CW cellulose deficient mutants (Additional file 8: Figure S5). All cellulose-deficient mutants tested displayed a significant reduction in vascular penetrations compared to their respective wildtype (WT) backgrounds—standard Col-0 for *ctl1-2*, *cobra-6*, *prc1-1* or Col-0 *JAZ10*_*pro*_*-GUS-Plus*^*sec*^ (JGP) for *cesa3-3*, and *kor1-4* (Figure 3A and B; Additional file 9: Tables S4A and S4B) [27]. No significant differences among the cellulose-deficient mutants themselves were observed (Figure 3A and B; Additional file 4: Tables S4A and S4B). To corroborate whether reduced vascular penetration events corresponded to reduced root colonization, we harvested surface-sterilized roots of Fo5176-infected plants and quantified fungal colonies originating from these roots. We detected a significant decrease in colony number from *ctl1-2*, *cobra-6*, and *prc1-1* plants compared to WT (Figure 3C). Similarly, we observed reduced colonies originating from *cesa3-3* and *kor1-4* plants compared to WT (Figure 3D).

To investigate the underlying transcriptional changes that contribute to the observed resistance in primary CW cellulose-deficient mutants, we conducted a transcriptomic analysis of 14 day-old WT and *ctl1-2* roots. These data have been deposited in the NCBI Gene Expression Omnibus [33] and are accessible through the GEO series accession number GSE168919. Differential gene expression analysis resulted in only 50 DEGs between the genotypes (adj. *p* value < 0.05, log_2_FC > |1|; Additional file 10: Table S5). Among them, we observed the upregulation of biological processes related with plant defense which were also activated in roots during Fo5176 infection: several peroxidases (PRXs), ET- and JA-related genes (Additional file 3 and 10: Supplementary Tables S2 and S5). Our transcriptomic data supports previously reported results of cellulose-deficient mutants exhibiting upregulation of defense response-related genes [46]. Taken together, these data suggest that plants impaired in primary CW cellulose synthesis could be “primed” for defense response activation, which we aimed to confirm further.

### Lignin deposition is a consequence of Fo5176 infection but is not essential for plant defense

**Fig. 4:**
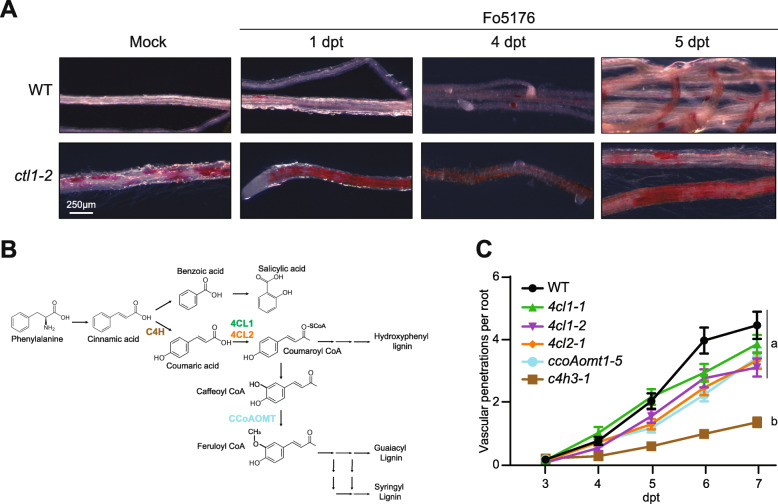
Ectopic lignification in cellulose-deficient mutants does not contribute to Fo5176 resistance. **A** Representative images of lignin deposition visualized by phloroglucinol stain (fuchsia) in WT (Col-0) and *ctl1-2* mock and Fo5176*-*infected roots at 1, 4, and 5 days post-treatment (dpt) with Fo5176 pSIX1::GFP microconidia. A minimum of 10 plants per genotype from at least 3 independent experiments were observed per dpt with similar results. **B** Schematic representation of the lignin biosynthesis pathway with focus on the bifurcation between phenylpropanoids and salicylic acid synthesis pathways. Mutants of biosynthesis enzymes in colored text are used in this study (see (**C**)). Figure adapted from [67]*.* **C** Root vascular penetration of WT (Col-0) and lignin-deficient mutants at various days post-treatment (dpt) with Fo5176 pSIX1::GFP microconidia. Values represent the mean ± standard error of at least 3 independent experiments, each one containing at least 13 seedlings. Statistical significance calculated via repeated measures two-way ANOVA with Tukey post-hoc test (*p* value ≤ 0.05 (genotype), *p* value ≤ 0.05 (time), *p* value ≤ 0.05 (genotype x time)). Significant differences at the last time point shown (7 dpt) are indicated on the graph using letters; statistics of remaining time points summarized in Additional file 9: Table S4A-F

Plants exposed to microbes have been reported to increase lignin deposition in their CWs to reinforce this structural barrier [25]. Our time course transcriptome revealed a significant upregulation of several early stage lignin biosynthesis genes from 3 dpt on: *phenylalanine ammonia-lyase 1* and 2 (*PAL1*, *2*) and *cinnamate-4-hydroxylase* (*C4H*) (Additional file 3: Table S2). In addition, PRXs implicated in lignin cross-linking at the CW were also upregulated in our transcriptome data set (Additional file 3: Table S2). Accordingly, we detected an increase in lignin deposition in Fo5176-infected WT roots starting at 4 dpt (Figure 4 A, upper panel). An upregulation of PRX genes was also found in *ctl1-2* seedlings (Additional file 10: Table S5). Therefore, the ectopic lignification associated with primary CW cellulose-deficiency in regions surrounding the plant vasculature could explain the resistance of these mutants to Fo5176 [24, 25, 47]. Indeed, *ctl1-2* roots exhibited increased deposition of lignin in response to Fo5176 already at 1 dpt (Figure 4A). To further understand the role of lignification during infection, we evaluated the response of lignin-deficient mutants to Fo5176*.* The mutants used—*4 cl1-1* and *4 cl1-2*, *c4h3-1*, and *ccoAomt1-5*—are impaired in various early stages in the lignin biosynthesis process and are all lignin-deficient (Figure 4B [48]). None of the lignin-deficient mutants tested exhibited less vascular penetration events than WT. Indeed, most of these mutants exhibited significantly reduced vascular penetration events only at 6dpt (Figure 4C; Additional file 9: Table S4C). Interestingly, *c4h3-1* mutants exhibited significantly reduced vascular penetrations compared to WT through the whole infection process. Our results indicate that Arabidopsis roots deposit lignin in their CWs in response to Fo5176 colonization, but lack of lignin synthesis does not impair plant defense against the fungus. Therefore, we conclude that ectopic lignin accumulation does not seem to account for the resistance observed in *ctl1-2* and other cellulose-deficient mutants.

### Callose deposition is not altered by Fo5165 infection

Together with lignin accumulation, reinforcement of plant CWs with callose deposition synthesized by *GLUCAN SYNTHASE–LIKE5* (GSL5) has been described as a host response to microbe colonization [49, 50]. As this polysaccharide also accumulates in mutants impaired in primary CW cellulose [51–53], we aimed to assess its role in Arabidopsis defense against Fo5176. In agreement with published data in tomato-Fo interactions [54], we did not observe significant differences in callose deposition between mock and Fo5176 infection (Additional file 11: Figure S6). Accordingly, our transcriptomic analysis did not reveal a significant change in *GSL5* expression during Fo5176 infection. Taken together, these data indicate that callose deposition does not play a major role in Arabidopsis-Fo5176 interaction.

### ET, but not JA signaling, induced by cellulose-deficiency contributes to Fo5176 resistance

**Fig. 5:**
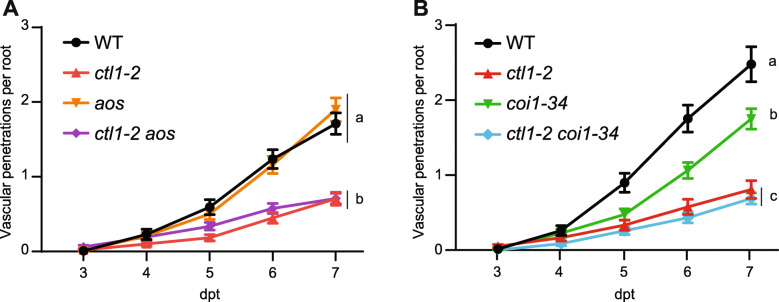
JA-mediated response does not contribute to the Arabidopsis resistance to Fo5176*.* **A**, **B** Root vascular penetration of JA biosynthesis (**A**) and signaling (**B**) mutants in WT (Col-0) and *ctl1-2* genetic backgrounds at various days post-treatment (dpt) with Fo5176 pSIX1::GFP microconidia. Values represent the mean ± standard error of at least 3 independent experiments, each one containing at least 10 seedlings. Statistical significance calculated via repeated measures two-way ANOVA with Tukey post-hoc test (*p* value ≤ 0.05 (genotype), *p* value ≤0.05 (time), *p* value ≤ 0.05 (genotype x time)). Significant differences at the last time point shown (7 dpt) are indicated on the graph using letters; statistics of remaining time points summarized in Additional file 9: Table S4A-F

Together with the described ectopic deposition of lignin, primary CW cellulose-deficient mutants have been reported to over-accumulate JA and ET compared to their WT counterparts [24, 47, 55]. Phytohormone-mediated signaling is absolutely imperative to proper defense response activation, and pre-existing enhanced accumulation of JA or ET could provide an explanation for enhanced disease resistance in primary CW cellulose-deficient mutants [7, 24, 26, 56]. Our time-course transcriptomic data indicated an upregulation of JA biosynthesis-related genes, including *ALLENE OXIDE CYCLASE* (*AOC) 1* and *2*, over the course of infection (Additional file 3: Table S2). These same genes were constitutively upregulated in *ctl1-2* compared to WT (Additional file 10: Table S5). Therefore, we tested whether JA-deficiency would negatively impact the resistance observed in cellulose-deficient mutants. We observed that the JA-biosynthesis mutant *aos* [57] demonstrated similar vascular penetrations compared to its WT control (Figure 5A; Additional file 9: Table S4D). We generated a *ctl1-2 aos* double mutant and observed that the *aos* mutation does not alter the resistance phenotype observed in *ctl1-2* (Figure 5A; Additional file 9: Table S4D). We then asked whether impairing JA-mediated signaling, but not biosynthesis, could explain the resistance observed in cellulose-deficient mutants. To this end, we made use of *coi1-34*, a weaker *COI1* allele [58]. We observed that *coi1-34* mutants exhibit less vascular penetrations than WT, while those of the *ct1-2 coi1-34* double mutant were not significantly different from *ctl1-2* at any time point (Figure 5B; Additional file 9: Table S4E). Therefore, our data indicate that neither increased JA biosynthesis nor signaling explain the resistance phenotype associated with cellulose-deficiency.

**Fig. 6:**
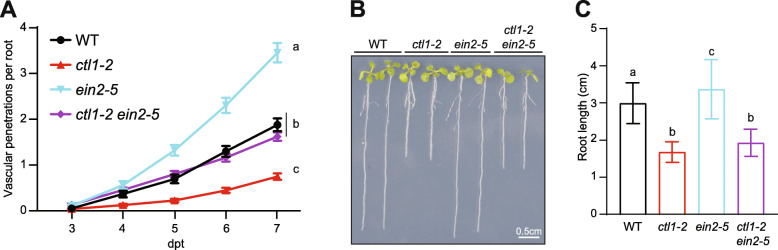
Upregulation of ET signaling contributes to the resistance of cellulose-deficient mutants during Fo5176 infection*.* **A** Root vascular penetration of the ET signaling mutant *ein2-5* in WT (Col-0) and *ctl1-2* genetic backgrounds at various days post-treatment (dpt) with Fo5176 pSIX1::GFP microconidia. Values represent the mean ± standard error of at least 3 independent experiments, each one containing at least 16 seedlings. Statistical significance calculated via repeated measures two-way ANOVA with Tukey post-hoc test (*p* value ≤ 0.05 (genotype), *p* value ≤ 0.05 (time), *p* value ≤ 0.05 (genotype x time)). Significant differences at the last time point shown (7 dpt) are indicated on the graph using letters; statistics of remaining time points summarized in Additional file 9: Table S4A-F. **B** Representative images of 8-day-old light-grown seedlings impaired in ET signaling in WT (Col-0) or *ctl1-2* background. **C** Quantification of root length of plants grown as depicted in **E**. Bars represent the mean ± standard deviation of *N* ≥ 40 plants averaged over three independent experiments. Statistical significance calculated via one-way ANOVA with Tukey post-hoc test (*p* value < 0.05)*.* Significant differences indicated using letters

We then asked whether upregulated ET signaling contributed to the observed Fo5176 resistance in *ctl1-2*. Transcriptomics analyses revealed that several ET response related genes were upregulated during Fo5176 infection, and one of them, *ERF94/ORA59*, was also constitutively upregulated in *ctl1-2* (Additional file 3 and 10: Tables S2 and S5). Based on these observed trends, we sought to understand the impact of impairing ET signaling in a cellulose-deficient background. To this end, we made use of the ET-signaling mutant *ein2-5* [59–61] and generated a *ctl1-2 ein2-5* double mutant. *ein2-5* displayed a significant increase in Fo5176 vascular penetrations compared to its WT and restored the *ctl1-2* resistance to WT levels, as the *ctl1-2 ein2-5* double mutant was as susceptible as WT to Fo5176 (Figure 6A; Additional file 9: Table S4F). Importantly, the *ctl1-2 ein2-5* did not suppress root growth stunting of *ctl1-2* plants (Figure 6B and C), demonstrating that shorter root length of primary CW cellulose-deficient mutants is not a contributing factor to their resistance to Fo5176. In addition, our results indicate that ET-mediated signaling plays a major role in Arabidopsis defense against Fo5176 root colonization and is a preeminent reason for the resistance to this fungus observed in *ctl1-2*.

### Cellulose-deficiency contributes to enhanced resistance to the vascular bacterial pathogen *Ralstonia solanacearum*

**Fig. 7:**
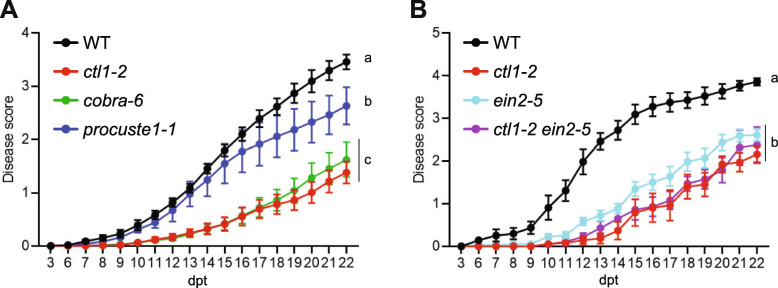
Cellulose-deficient mutants exhibit enhanced resistance to the bacterial pathogen *Ralstonia solanacearum.* **A**, **B** Disease scoring of cellulose-deficient mutants (**A**) and the ET signaling mutant *ein2-5* in WT (Col-0) and *ctl1-2* genetic backgrounds (**B**) at various days post-treatment (dpt) with the vascular bacteria *R. solanacearum* GMI1000*.* Qualitative data represented as line plots represents the average of at least 3 independent experiments, each one including ≥ 24 plants per genotype. Based on plant symptoms on each day, an average disease score was calculated per time point represented in line graphs. The disease scoring index measured symptoms on a scale of 1 to 4 (0 = no wilting, 1 = 25% wilted leaves, 2 = 50%, 3 = 75%, and 4 = death) as described in the methods. Statistical significance based on absolute number of plants assigned to either the least (≤ 1) or most (> 3 ≤ 4) diseased category calculated via Fisher’s exact contingency test indicated in Additional file 12: Table S6 A-G

Downregulation of cell wall-related processes has also been observed in early infection stages of Arabidopsis colonization by the bacterial root vascular pathogen, *Ralstonia solanacearum* [62]. Consistent with observations during Fo5176 pathogenesis, the primary CW cellulose-deficient mutants *ctl1-2*, *cobra-6*, and *prc1-1* exhibited significantly increased resistance to *R. solanacearum*, as these mutants showed lower disease scores compared to WT (Figure 7A; Additional file 12: Table S6A). We sought to understand whether ET signaling also contributes to the resistance conferred by cellulose-deficiency during *R. solanacearum* infection. We observed that the *ein2-5* mutant was significantly more resistant than WT to *R. solanacearum* (Figure 7B, Additional file 12: Table S6B). Furthermore, no significant difference was observed in the infection phenotypes of *ctl1-2* compared to *ctl1-2 ein2-5* double mutants (Figure 7B, Additional file 12: Table S6B). Our data indicate that the cellulose alterations in primary CWs contribute to broad disease resistance against root vascular pathogens, and in resistance to Fo5176 this is due to a role of ET signaling.

## Discussion

In this study, we provide detailed information about the intricate processes which govern root immune responses to Fo5176 using a multi-faceted approach. The intercellular infection strategy used by Fo to advance towards the root vasculature makes it an ideal candidate for understanding host-pathogen interactions in the apoplast with a focus on cellulose-rich plant primary CWs. Our dual transcriptomics approach, based on the characterization of Fo5176 root infection strategy via confocal microscopy, allowed us to provide spatial and temporal resolution of plant root and fungal genes involved in the Fo5176-Arabidopsis interaction. We observed significant and rapid downregulation of CW-related genes during the early stages of fungal proliferation in the apoplast, particularly of those related with primary CW-cellulose biosynthesis. Due to the importance of this polysaccharide in plant root biology, we focused on characterizing defense response in the corresponding cellulose-deficient mutants. Our experimental data allowed us to conclude that downregulation of the primary CW-cellulose synthesis machinery vastly reduces the capacity of Fo5176 to reach the root vasculature due to the upregulation of ET-signaling in primary CW mutants.

### Dual transcriptomics reveals an important role for cell wall-related genes during Fo infection

The use of dual transcriptomics allows for the simultaneous study of microbe and host acclimation to the interaction at the gene expression level. Our time course analysis spans the Fo5176 infection process from microconidia adhesion and germination at the root surface to xylem colonization (Figure 1).

Our dataset showed that Fo5176 regulates its transcriptomic profile while it invades the root. Over time, an increasing amount of genes show upregulated expression compared to the fungal microconidia germinated *in vitro*. We identified more than 400 DEGs continuously upregulated or downregulated in planta compared to in vitro at every investigated time point (Additional file 4: Table S3; Clusters 1, 2, and 5 in Additional file 6: Figure S3). These genes seem to be responsible for the fungal acclimation to the host and are not necessarily connected to changes in the fungal lifestyle in the different root layers. Most of the CW modifying genes co-expressed following a non-homogenous expression pattern through the root layers: they were upregulated until the first xylem colonization events (4dpt) and then their expression decayed (Additional file 4: Table S3; Cluster 3 in Additional file 6: Figure S3). This significant activation of the fungal CW modification machinery in the cell root layers that precedes the vasculature coincides with the need of the microbe to pass through the plant primary CWs. The fungal hyphae have to switch from a nutrient-rich growth in PDB to a nutrient-poor situation in plants. Using the CW modifying artillery, Fo could improve the availability of nutrient resources from plant CWs as well as restructure its own CW to enable root colonization.

The fungus does not cause a significant transcriptional reprogramming of the host while it stays at the root surface (1dpt), reflected in no significant transcriptional reprogramming compared with mock treated plants. A clear host response to Fo5176 started at the early time point of 2 dpt, when the epidermal apoplast was invaded. The downregulation of primary CW-cellulose synthesis was one of the first responses of the plant to the pathogen, while we could not detect a strong activation of defense mechanisms at this time point ([63]; Figure 2B; Additional file 3: Table S2). From 3dpt on, the expression of defense-related genes significantly increased, like the JA- and ET-responsive defensin PDF1.2 [64] and the NADPH oxidase RBOHD required for ROS production during innate immunity [63] (Figure 2B; Additional file 3: Table S2). This corresponds to the time point when the fungal hyphae reached the cortex layer (Fig. 1A) [31]. Our data suggest that the root is not significantly affected until the microbe reaches the cortex, in agreement with recent work showing that the root epidermal cell layer responds to microbes only when a certain threshold of damage has been exceeded [65]. Time-course transcriptome analysis revealed that the mechanism by which roots respond to Fo5176 infection is an evolutionary process that transitioned from growth inhibition to active defense. Our data expands and defines more precisely available studies on Arabidopsis root transcriptional reprogramming upon Fo5176 infection [12, 13] (Additional file 13: Table S7).

### Fo5176-resistance in primary CW cellulose-deficient mutants can be eliminated by blocking ET signaling

We observed downregulation of primary CW *CESA* genes at the early Fo5176 infection stages corresponding to hyphal penetration into the epidermal layers (Figure 1A). This response could be induced by the fungus to weaken host cell walls or could be a response from the plant to temporarily pause developmental growth to favor resource allocation towards defense response. Our work demonstrates that, despite their weakened cell walls, plants with reduced primary CW-cellulose are more resistant to root vascular pathogens. We initially observed that the primary CW-cellulose deficient mutants *ctl1-2*, *cobra-6*, and *prc1-1*, *cesa3-3* and *kor1-4* were all more resistant to Fo5176 in terms of reduced vascular penetration and fungal colony counting than their respective WTs (Figure 3)*.* To better understand the broad-spectrum effects of primary CW-cellulose-deficiency on defense response, disease phenotypes upon infection with the vascular bacterial pathogen *R. solanacearum* were also evaluated*.* This pathogen is the causative agent of bacterial wilt, and its infection strategy has been well-established to involve modification and degradation of the plant cell wall [46, 66]. Consistent with our observations of Fo5176 infection, a clear trend towards resistance was observed in *ctl1-2*, *cobra-6*, and *prc1-1* compared to WT (Figure 7A). Our results suggest that mutation of primary cell wall genes, including *CESAs*, also confers enhanced resistance to root vascular pathogens of different kingdoms.

A connection between primary CW-cellulose-deficiency and biotic resistance has been hypothesized on, among others, the ectopic deposition of lignin and callose shown by the corresponding plant mutants [24]. Lignin deposition contributes to plant resistance to foliar bacteria [25]; however, deposition of lignin in roots during Fusarium infection has not been demonstrated. Our data confirmed the reinforcement of the WT root CWs with lignin in response to Fo5176 infection (Figure 4 A) as a consequence of the upregulation of lignin biosynthesis and deposition-related genes from 3 dpt on (Additional file 3: Table S2). Conversely, our data indicates that callose deposition is not altered by Fo5176 infection (Additional file 11: Figure S6).

We observed that most lignin-deficient mutants tested do not exhibit enhanced susceptibility to Fo5176 compared to WT (Figure 4C). On the contrary, all of them showed increased tolerance to the fungus at 6dpt that disappeared at 7dpt, with the exception of *c4h3-1* that was significantly more resistant than WT throughout the whole experiment. This mutation interrupts the lignin biosynthesis pathway at its bifurcation with the SA biosynthesis pathway (Figure 4B). A mutant lacking C4H may accumulate an increased pool of cinnamic acid, thereby leading to its conversion to benzoic acid by an alternate pathway that would eventually result in increased SA synthesis, as demonstrated by chemical inhibition of C4H [67]. Therefore, it is possible that SA-mediated signaling could contribute to the resistance phenotype to Fo5176 observed in *c4h3-1* mutants. Another possibility for the increased resistance to Fo5176 of plants with altered lignin content and composition is their ectopic expression of CW degrading enzymes that released CW fragments acting as signals for defense activation [68, 69]. Our data indicate that root lignification is a programmed response to Fo5176, but is not an essential physical barrier to block fungal advance. In addition, *ctl1-2* mutants increased the amount of lignin in the roots in response to Fo5176 much earlier than the WT (1dpt vs 5dpt; Figure 4A). These observations indicate that WT plants need to activate resources in order to achieve this lignification only upon successful perception of the fungus, while the lignin deposition machinery of *ctl1-2* plants is readily activated upon Fo5176 detection. We observed increased expression of genes encoding for peroxidases, *PRX37* and *PRX52*, in *ctl1-2* compared to WT, while the expression of lignin biosynthesis genes was not altered in *ctl1-2* (Additional file 10: Table S5). PRX37 and PRX52 are involved in lignin deposition and their transcripts were upregulated in WT roots over the course of Fo5176 infection [70, 71]. Overall, our data indicates that ectopic lignification in *ctl1-2* and its rapid over-lignification response to Fo5176 is likely influenced by the activity of PRXs and other late-stage lignin biosynthesis proteins.

Our data corroborated previous reports that connect modifications in primary cell wall cellulose with differential regulation of JA/ET-related genes in *ctl1-2* (Additional file 10: Table S5) [24, 29, 72]. Moreover, the connection between hormone regulation and defense responses pertaining to vascular pathogens, such as Fo5176, was strengthened in this study by identifying the upregulation of hormone-related genes during infection (Additional file 3: Table S2) [73–75]. It was previously demonstrated that *F. oxysporum* specifically hijacks non-defense response-related aspects of JA signaling mediated by COI1 to induce Arabidopsis infection [76]. This host manipulation led to an enhanced wilting resistance observed in the JA-signaling mutant *coi1*, but not in the synthesis mutant *aos* [8, 76]. Accordingly, Arabidopsis *coi1* exhibited reduced susceptibility to the soil-borne bacterial pathogen *R. solanacearum* [66, 76]. Importantly, the Arabidopsis *coi1*-mediated resistance to Fo was only associated with late stages of the infection (approximately 21 dpt) but not with reduced fungal penetration and xylem colonization, as similar amounts of Fo were detected in the mutant and the wild-type [76]. Our data confirmed this result and further expanded it, as we showed that neither JA biosynthesis nor signaling influence the ability of Fo5176 to reach the xylem in primary cell wall cellulose-deficient mutants (Figure 5).

Based on the observations that neither the ectopic lignification nor defects in JA signaling could fully explain the enhanced resistance observed in primary CW-cellulose-deficient mutants, we then investigated the role of ET signaling. It was previously suggested that ethylene response factor 1 (ERF1), a downstream component of the ET and JA pathways, acted as a positive defense response signal in the context of Fo infection using a spray infection method [45, 73]. Our data confirm previous studies showing that ET-dependent genes are activated as the primary response during pathogen response activation prior to JA-mediated signaling [77, 78]. We observed upregulation of genes encoding ET responsive transcription factors (*ERF1*, *62*, *73*, *94* and *113*) during Fo5176 infection from 2 dpt on, while JA-responsive genes were significantly upregulated only at 3 dpt on, including several *JAZs* (Additional file 3: Table S2). Additionally, ET-responsive transcription factor *ERF94* was constitutively expressed in *ctl1-2* plants compared to WT (Additional file 10: Table S5)*.* We observed that blocking ET signaling impaired plant defense against Fo5176 and was sufficient to disrupt *ctl1-2*-associated resistance phenotypes (Figure 6). The upregulation of ET-related genes in roots infected by Fo5176 and their constitutive expression in *ctl1-2* suggest that the WT-like resistance of *ctl1-2 ein2-5* might be due to a direct suppression of the *ctl1-2* effect by *ein2-5*. However, an additive action of two independent resistance pathways, CTL1- and EIN2-mediated, with opposite roles in plant defense to Fo5176 cannot be completely discarded. An additional explanation for the general resistance of cellulose-deficient mutants to Fo5176 could simply be due to their shorter root length. As previously reported, all the primary CW cellulose-deficient mutants tested in this study display the classic primary CW cellulose-deficient dwarfed root phenotype [19, 23, 79, 80]. It could be more difficult for the fungus to reach shorter roots or to find an area of weakness for penetration due to lack of available root space. Conversely, impairing ET-signaling did not alter the dwarfed root phenotype of *ctl1-2*, but restored the observed Fo5176 resistance (Figure 6), thus negating the possibility that root length is a contributing factor to the enhanced defense response in *ctl1-2.* Our data strongly support a role for ET-dependent but JA-independent root defense against Fo5176. We observed that impairing ET signaling in the context of primary CW-cellulose-deficiency eliminates the observed resistance phenotype to Fo5176. Interestingly, our data indicate that ET-mediated signaling plays a major role in Arabidopsis defense against *R. solanacearum*, but it does not seem to account for the resistance observed in cellulose-deficient mutants (Figure 7B). This observation indicates that primary CW-cellulose defects increase plant defense to various root vascular pathogens, but the molecular mechanisms underlying these resistant phenotypes are heavily dependent on the pathosystem. Our observations ultimately suggest an important role for hormones, specifically ET, in enhanced disease resistance in primary CW-cellulose-deficient mutants.

## Conclusions

In this study, we show how plant roots and their vascular pathogens tightly control their gene expression dynamics during their interaction. Our time-resolved dual transcriptomic approach represents a useful tool to identify root and fungal molecular players implicated in the infection process. Specifically, our findings reveal a critical role of rapid reduction of primary CW cellulose synthesis in plant defense against root vascular pathogens. Furthermore, we highlight a novel role for ET signaling as a molecular basis to compensate for the resistance associated with primary CW cellulose-deficiency during Fo5176 infection, which is surprisingly JA-independent. In summary, we shed light on the complex interaction between hormone-mediated signaling, root CW composition, and defense response activation

## Methods

### Arabidopsis growth conditions

*Arabidopsis thaliana* accession Col-0 plants were grown under long-day conditions (16 h light, 8 h dark) with optimized light intensity (130–150 μE m^−2^ s^−1^) at 20–22 °C, unless otherwise indicated. Seeds used for all in vitro experiments were either gas or liquid-sterilized and grown on ½ MS media (Duchefa; catalog number M0222.0025) supplemented with 1% (w/v) sucrose (when indicated) and 0.9% bacteriological grade agar (Difco; catalog number 214530). For hydroponic experiments, Arabidopsis seeds were germinated at 24 °C, long day conditions, on 2 mm foam plugs suspended on 200 ml ½ MS + 1% sucrose media in 330 ml pots at pH 5.7 adjusted by KOH. The media was exchanged 6 days after germination to ½ MS and seedlings were further grown.

Mutant genotypes were confirmed using primers previously reported or designed for this work (Additional file 14: Table S8): *ctl1-2* (SALK_093049) [21, 79], *cobra-6* (SALK_051906) [53], *procuste1-1* [19, 20], *kor1-4* [27], lignin-deficient mutants (*4 cl1-1*, *4 cl1-2*, *4 cl2-1*, *ccoAomt1-5*, *c4h3-1*) [81], *aos* (*CYP74A*) [57], *coi1-34* [82], and *ein2-5* [60, 83]. The *cesa3-3* allele was identified as a negative regulator of JA signaling in a forward genetic EMS screen. The mutant allele exhibited ectopic expression of the JA-responsive reporter *JAZ10p:GUSPlus* and increased *JAZ10* transcript levels in both shoots and roots. The causative GAT to AAT transition leading to a D378N mutation underlying the *cesa3-3* phenotype was identified via mapping-by-whole-genome-sequencing of bulk segregants as described and confirmed by allelism tests with the characterized *cesa3* mutant allele *cev1* [28, 29].

The *cesa3-3* allele was identified as a negative regulator of JA signaling in a forward genetic EMS screen [82]. The mutant allele exhibited ectopic expression of the JA-responsive reporter JAZ10p:GUSPlus and increased JAZ10 transcript levels in both shoots and roots. The causative Gat to Aat transition leading to a D378N mutation underlying the *cesa3-3* phenotype was identified via mapping-by-whole-genome-sequencing of bulk segregants as described [82] and confirmed by allelism tests with the characterized *cesa3* mutant allele *cev1* [29]. Primary root length and width were evaluated in 7-day-old seedlings as described [27, 82].

### Fungal constructs, growth, and infection assays

To obtain the Fo5176 pGPD::GFP line, the previously reported pPK2-*hphgfp* construct containing a Hygromycin resistance-GFP fusion protein under the control of the constitutive gpdA promoter (pGPD) [84] was inserted into Fo5176 by *Agrobacterium* mediated transformation as described before [85].

Fo5176 growth and in-plate infection assays were conducted as previously described [11, 86]. Briefly, sterilized Arabidopsis seeds were sown on sterilized Whatman paper strips (VWR International, catalog number 514-8013) placed on top of the media as described above. Plants were grown vertically in long-day conditions for 8 days and infected as previously reported [11, 86]. Vascular penetrations were counted using fluorescence stereomicroscopy and root length measurements were quantified via scanned images. Infection time-points are referred to as “days post-treatment” (dpt). Statistical analyses were conducted in GraphPad Prism 8 (version 8.4.3).

For hydroponic infections, the roots of 10 day-old seedlings were infected with 20 μl of a solution containing 10^7^ microconidia/ml Fo5176. All pots were incubated for 30 min at 100 rpm on a rotary shaker. The media of the infected plants was then replaced with fresh ½ MS media. The plants were further grown under the same conditions until the roots were harvested for RNA extraction or for imaging at 0, 1, 2, 3, 4, and 6 days post treatment.

For Fo5176 transcriptomics in the absence of a plant host, 10^7^ microconidia/ml were germinated in ½ MS + 1% (w/v) sucrose overnight at 180 rpm at 28 °C in the dark. The germinated microconidia were harvested via two centrifugation steps at 4000×*g* for 15 min at 10 °C, washed twice with water, discarding supernatant in between washes. The pellet was frozen in liquid nitrogen (LN_2_) and subsequently freeze-dried. The lyophilized pellet was used for RNA extraction.

For colony quantification after Fo5176 infection, roots of infected plants were harvested at 7 dpt and weighed. Roots were surface sterilized for 1 min in 80% ethanol (alcosuisse), followed by 1 min in 0.25% sodium hypochlorite (Chemie Brunschwig AG), lastly followed by three 1 min washes in sterile water. The water from the final wash was collected as a sterilization control. Four to 10 sterile glass beads (2.85–3.45 mm diameter, Carl Roth GmbH + Co, Germany) were added to tubes and washed/sterilized root material was then ground in 1 mL of sterile water using a GenoGrinder (Retsch MM301, Retsch GmbH + Co, Germany) for approximately 3 min at maximum speed. One hundred microliters of the sterilization control and 1 mL of ground root material were plated separately on ½ Potato Dextrose Broth (BD Difco, catalog number: 0549-17-9) + 1% agar plates (BD Difco, catalog number: 214530) supplemented with 25 μg/ml chloramphenicol (Sigma Aldrich, catalog number: C0378) and 55 μg/ml hygromycin (Sigma Aldrich, catalog number: H9773). Plates were then sealed with parafilm and incubated at 28 °C. Fungal colonies were quantified after 3 days of incubation. Colony quantifications were normalized to root fresh weight (mg).

### *Ralstonia solanacearum* growth and infection assay

*R. solanacearum* pathogenicity tests were carried out using the soil-drench method as previously described [87]. Briefly, Arabidopsis was grown for 4 to 5 weeks on Jiffy pots (Jiffy Group, Lorain, OH, U.S.A.) in a controlled chamber at 22 °C, 60% humidity, and an 8-h light and 16-h dark photoperiod. Three vertical holes were made in Jiffy pots, and the pots were immediately submerged for 30 min into a solution of overnight-grown *R. solanacearum* strain GMI1000 adjusted to OD_600_ = 0.1 with distilled water (30 mL of bacterial solution per plant). Inoculated plants were then transferred to trays containing a thin layer of soil drenched with the same *R. solanacearum* solution and were kept in a chamber at 27 °C, 60% humidity, and 12 h of light and 12 h of dark. Plant wilting symptoms were recorded every day and were expressed according to a disease index scale. The disease index measured symptoms on a 1 to 4 scale (0 = no wilting, 1 = 25% wilted leaves, 2 = 50%, 3 = 75%, and 4 = death). Infection time points associated with soil-drenching infection are referred to as “days post-inoculation” (dpt). Statistical analyses were conducted in GraphPad Prism 8 (version 8.4.3).

### Confocal imaging

Arabidopsis Col-0 seedlings were infected with the fluorescently labeled strain Fo5176 pGPD::GFP, placed on chambered cover glasses (Thermo Scientific™ Nunc™ Lab-Tek™) and covered with thin blocks of solid ½ MS medium. Images were taken with a Zeiss LSM 780 Axioobserver microscope, using the LD C-Apochromat 40x/1.1 W Korr M27 objective and Immersol W (Zeiss) between lens and coverslip. GFP (fungus) was excited at 488 nm and emitted fluorescence was detected at 514 nm. RFP (Arabidopsis autofluorescence) was excited at 561 nm and emission was detected at 641 nm, being the Pinhole for both channels 36.28 um. Z-stacks of individual roots were obtained by imaging every 1.91 um to obtain a transversal optical section (Zen Lite 2012).

### RNA extraction and sequencing

For the dual time course transcriptome analysis, roots from 2 pots were pooled for each condition and time point separately for RNA-extraction. Roots were harvested by manual removal from foam plugs and were dried gently using tissue paper. Roots were weighed and immediately frozen in liquid N_2_. The root samples were stored at − 80 °C until RNA was extracted. Four replicates were harvested per time point for infected and control plants and 2 replicates for the in vitro grown microconidia.

For the RNA-Seq of *ctl1-2* and WT (Col-0) plants, roots from 14-day-old seedlings grown on plates as described above were harvested and immediately frozen in LN_2_. Three independent replicates were used for the transcriptome assay.

Root material or germinated microconidia were ground with mortar and pestle in LN_2_. Fifty to one hundred milligrams of ground material was used for RNA extraction using the RNeasy plant mini Kit (Qiagen). Extraction was performed according to the user manual provided by the supplier (RNeasy Mini Kit handbook, Fourth edition, June 2012, Qiagen). Extraction buffer RLC with 10 μl/ml β-mercaptoethanol freshly added was used to extract the RNA. An on-column DNA digestion (RNase-Free DNase Set, Qiagen) was performed. Before elution of the RNA the column was incubated 1 min with 30 μl RNAse-free water to resolve the RNA. Concentration of the RNA was determined by Nanodrop (Thermo Fisher) and integrity of the RNA was examined by gel electrophoresis on a 1% agarose gel. For samples with low concentration, the RNA content was additionally measured by Qubit Fluorometer using the RNA BR assay kit (Qubit, Thermo Fisher).

3′mRNA-libraries were prepared using the 3′mRNA-Seq library Prep Kit (QuantSeq, Lexogen). The manual from the supplier was followed with the following modifications. At least 1 μg RNA was used as input. For first strand synthesis of cDNA, the incubation time at step 4 was increased to 60 min. For library indexing and amplification, 13–17 amplification cycles of the given PCR were used depending on the amount of input RNA. Seventeen microliters of the purified library was transferred to a fresh tube. The finished libraries were stored at − 20 °C until quality control and pooling. The quality of the libraries was assessed with D1000 ScreenTape on Agilent 4200 Bioanalyzer at the Functional Genomic Center Zürich (FGCZ) with the included software from the manufacturer. Libraries were pooled equi-molecularly for sequencing.

Sequencing of the time course experiment was performed in an Illumina HiSeq2500 sequencer (single-end 125 bp) with a read depth of around 5 mio reads per sample. Samples were divided in three charges and sequenced in different runs of the sequencer. Sequencing of the *ctl1-2* vs WT experiment was performed in an Illumina NovaSeq6000 sequencer (single-end 100 bp) with a read depth of around 8 mio reads per sample. Resulting reads were assigned to the samples based on their index-number by FGCZ. Raw reads separated by sample were obtained in FastQ-format from FGCZ.

### Read mapping

Read mapping and quantification was performed on SUSHI (FGCZ) [88]. The reads were trimmed (Trimmomatic, version 0.36) [89] and adapters were removed (Flexbar, version 3.0.3) [90]. Reads > 20 bp were kept. Reads were mapped by STAR (version 2.6.1c) [91] against the *Arabidopsis thaliana* genome (TAIR10 [32]) and *Fusarium oxysporum* Fo5176 [10]. Uniquely mapped reads were counted by featureCounts [92], based on the R package Rsubread (version 1.32.1) [93].

### Differential expression analysis and co-expression analysis of DEGs

Differential expression analysis was conducted using the Bioconductor package edgeR (version 3.24.3) [94]. The raw read-counts were imported to EdgeR and counts were normalized using trimmed means of M-values (TMM) normalization [95]. Genes with ≥ 3 counts per million (CPM) were assigned as actively transcribed genes. Dispersion was estimated using the quantile-adjusted conditional maximum likelihood (qCLM) method. Differential expression was computed using glmTREAT, *p* value was adjusted using Benjamini-Hochberg correction.

Significantly differentially expressed genes (DEGs) were considered those with aLog_2_ fold-change > |1| and a FDR < 0.05 and were used for clustering expression profiles along the time points of the experiment (Mfuzz version 2.46.0, Bioconductor) [34].

### Gene functional analysis

Gene ontology (GO) term enrichment for Arabidopsis was performed in cluster-wise manner with ClueGO (version 2.5.7) [96] in Cytoscape (version 3.7.0) [97]. A list of Arabidopsis cell wall related genes was generated based on the data available on the Cell Wall Genomics database (https://cellwall.genomics.purdue.edu/, 24 March 2020). A list of Arabidopsis transcription factors, carbohydrate active enzymes, hormone-related genes, and hormone biosynthetic genes was obtained from [98]. A list of Fo5176 carbohydrate active proteins was generated using the online service dbCAN2 with HMMER, DIAMOND and Hotpep tools. Genes with a Carbohydrate Active enZYme (CAZY) -domain prediction by at least 2 tools were considered as CAZY-domain containing genes (http://bcb.unl.edu/dbCAN2/, 8 October 2020 [99]).

### qRT-PCR validation of DEGs

Control and infected samples of 4 additional biological replicates were used for validation of the DEGs. Root material was ground by mortar and pestle in LN2. One milliliter TRI Reagent for DNA, RNA, and protein isolation (Sigma-Aldrich, Merck, Germany) was added and mixed thoroughly by hand. Tubes were incubated 5 min slowly shaking at room temperature and 0.2 ml chloroform was added. Tubes were shaken manually for 15 s, followed by 3 min incubation at room temperature. Samples were centrifuged at 4 °C, 12000×*g*, 15 min; 400 μl of the supernatant was transferred to fresh tubes. Five hundred microliters of isopropanol (4 °C) was added; the tubes were inverted three times and incubated for 10 min on ice followed by 15 min centrifugation at 4 °C, 15000×*g*. Supernatant was removed and pellets were resuspended in 1 ml 75% ethanol (4 °C), mixed by vortexing and centrifuged for 5 min at 4 °C, 7500×*g*. Supernatant was removed and samples were air-dried on ice for 10 min. Pellets were resuspended in 45 μl of RNAse-free water. Five microliters of RNA was kept on ice to measure concentration and evaluate integrity as described above. The remaining 40 μl were frozen in LN2 and kept at − 80 °C until cDNA-synthesis.

cDNA synthesis was performed using Maxima H Minus cDNA-synthesis-kit (Maxima H Minus First Strand cDNA Synthesis Kit, with dsDNase; Thermo-Fisher) according to the instructions of the supplier. One microgram of RNA was used in each reaction as a template.

qRT-PCR was performed on a LightCycler 480 (Roche), using Fast SYBR Green Master Mix (ThermoFisher Scientific). Four microliters 1:16 diluted cDNA, 5 μl of SYBR Green Master Mix, and 0.5 μl of each primer (Additional file 14: Table S8) was prepared in a 384-well plate. The qRT-PCR-program was set as follows: initial denaturation for 3 min, 95 °C; 50 cycles of denaturation at 95 °C, 15 s, annealing at 60 °C, 10 s, elongation at 72 °C, 15 s; final elongation at 72 °C, 30 s; melting curve of 0.11 °C/s temperature increase from 42 °C to 95 °C; cooling down to 20 °C, 30 s. Two control sets of primers were used for Arabidopsis GAPDH 3′-end and 5′-end [100]. The mean of the two reference transcripts were used for normalization of signal for the other genes tested. Fo5176 β-Tubulin (Fo5176.g4360) was used as a reference gene for *Fusarium oxysporum* gene expression. For the calculation of the expression the 2^−ΔΔCt^ method was used [101], and the fold changes in expression were represented as the ratio of the mean value of infected/mean value of control Ct.

Expressions of selected DEGs obtained by RNA-Seq and qRT-PCR were compared to each other using the log_10_-transformed fold change by Pearson correlation (GraphPad Prism version 9.0.2 (161)).

### Cellulose quantification

Roots of 10 day-old seedlings grown as indicated under “Arabidopsis growth conditions” were harvested and processed as described before to measure the crystalline cellulose [11, 102].

### Lignin staining

Seven-day-old WT, *cesa3-3*, and *cev1* seedlings or plants infected as described above (“Fungal constructs, growth, and infection assays”) at the indicated time points were incubated for 10 min with soft agitation in phloroglucinol stain prepared as follows: 25 mg phloroglucinol (Brunschwig, catalog number ACR24176-0250), 25 mL 37% hydrochloric acid (Sigma Aldrich, catalog number 30721-1 L), and 25 mL methanol. Phloroglucinol stain was removed using a plastic pipette and replaced with a 3:1 mixture of glycerol to water. Plants were placed on plates containing ½ MS media + 1% sucrose and immediately imaged using a stereomicroscope. For all days, the images for WT and *ctl1-2* were taken with the same settings. Because of their cellulose deficiency, *ctl1-2* cells are bulged and the roots are thicker than WT ones; thus, *ctl1-2* images are darker than WT ones. Therefore, we modified the brightness and contrast of the images to improve the visualization of the lignin staining and obtain a better visual precision and accuracy.

### Callose staining

Plants infected as described above (in the “Fungal constructs, growth, and infection assays” section) were collected at 7dpt and stained for callose as described in [103].

## Supplementary Information

**Additional file 1: Table S1**. Overview of read counts mapping to Arabidopsis and Fo5176. (Sheet 1) Read mapping overview of sequencing libraries mapped to the Arabidopsis reference genome (TAIR10, [32]**)** or the Fo5176 reference genome [10]. (Sheet 2) Arabidopsis and Fo5176 mapped genes with ≥3 CPM (counts per million). Genes with < 3 CPM in all samples were excluded from differential gene expression analysis. (Sheet 3) Read mapping overview of sequencing libraries of *ctl1-2* and WT (Col-0) mapped to the Arabidopsis reference genome (TAIR10, [32]) and overview of genes detected with CPM ≥ 3 compared to all genes used for analysis.

**Additional file 2: Figure S1**. Complete MDS-Analysis of Arabidopsis transcriptional profiles. Multidimensional scaling analysis (MDS) of transcriptional profiles of Arabidopsis. Samples clearly deviating from the rest of the samples at the same time point and condition (surrounded by a red square) were not used for further analysis.

**Additional file 3: Table S2**: Arabidopsis differentially expressed genes (DEGs) in response to Fo5176 infection over time. (Sheet 1) Significant DEGs in Arabidopsis roots upon infection with Fo5176 at different time points after treatment (dpt) with Fo5176 microconidia compared to mock-treated roots. Logarithmic fold-change (logFC) and corrected *p*-value (FDR) are presented in columns for each time point separately, gaps correspond to non-significant changes in gene expression at those time points. The genes are clustered based on their co-expression (Figure 2A). DEGs with the entry “no cluster” were not associated to a cluster by the clustering algorithm. Genes encoding proteins containing a CAZY-domain or related with plant cell wall biology are highlighted in yellow and orange, respectively. (Sheet 2) GO-enrichment analysis of the clusters from (Sheet 1). GO-ID: Gene ontology identifier, GO-term: Gene ontology descriptive term, Bonferroni-corrected *p*-value, % of associated genes: percentage of genes in that GO-term that are present in the analyzed cluster, No. of genes: absolute number of genes associated to the GO-term.

**Additional file 4: Table S3**. Fo5176 differentially expressed genes (DEGs) during Arabidopsis root infection over time. Significant DEGs in Fo5176 during Arabidopsis root infection compared to *in vitro* germinated microconidia. Logarithmic fold-change (logFC) and corrected *p*-value (FDR) are presented in columns for each time point separately; gaps correspond to non-significant changes in gene expression at those time points. The genes are clustered based on their co-expression pattern (Additional file 5: Figure S2A). DEGs with the entry “no cluster” were not associated to a cluster by the clustering algorithm, DEGs only expressed in vitro were not included in the clustering (excluded). All DEGs were further analyzed for presence of carbohydrate active domains in their corresponding encoded protein sequences using the dbCAN2-meta server (http://bcb.unl.edu/dbCAN2/, 8 October 2020) and were highlighted in yellow (Additional file 5: Figure S2B). The genes were described based on IPR and PFAM; IPR_description: protein family classification by InterPro, PFAM: protein family classification by PFAM (both obtained from [10]).

**Additional file 5: Figure S2**. RNAseq validation by qRT-PCR. **(A)** and **(B)** The expression of 6 randomly picked DEGs from Arabidopsis (A) or Fo5176 (B) expressed at different levels based on the RNA-sequencing (left panels) was confirmed by qRT-PCR (right panels) using *de novo* generated RNA samples. The qRT-PCR-based expression of each gene was determined relative to the corresponding reference gene; i.e. At GAPDH in (A) and Fo5176 β-Tub in (B). **(C)** and **(D)** Correlation (log_10_ fold changes) between RNAseq and qPCR derived expression data from Arabidopsis (A and C) and Fo5176 (B and D). Very good Pearson correlations of *r* = 0.79 for Arabidopsis and *r* = 0.97 for the fungus were obtained (*p*-value < 0.0001 in both cases). Linear trend lines are depicted in black.

**Additional file 6: Figure S3**. Temporal dynamics of Fo5176 DEGs during root infection reveal a significant alteration of proteins containing catalytic and carbohydrate-binding modules (CAZY). **(A)** Clusters of Fo5176 coexpressed DEGs during infection using fuzzy c-means clustering. **(B)** Number of DEGs encoding proteins with carbohydrate active enzyme-domains (CAZY) and glycosyl hydrolases in the different clusters. DEGs with the entry “no cluster” were not associated to a cluster by the clustering algorithm.

**Additional file 7: Figure S4**. *ctl1-2* is impaired in crystalline cellulose deposition in the root. Cellulose content of roots from 10 day-old light-grown plants represented as ug of D-glucose derived from crystalline cellulose per mg of dried alcohol-insoluble residue (AIR) [102]. *N* = 2 biological replicates +/- standard deviation; 3 technical replicates per biological replicate. Welch’s unpaired t-test; ***P-value = 0.0004.

**Additional file 8: Figure S5**. Characterization of *cesa3-3*. **(A)** Representative images of 7 day-old WT, *cesa3-3* and *cev1* seedlings. **(B)** Primary root length box plots of indicated genotypes. Medians are represented inside the boxes by solid lines, and circles depict individual measurements (*n* = 38-61). (**C)** Representative primary root images specifying the initiation of the differentiation zone by the appearance of root hairs (dashed line), and bulging cells in *cesa3-3* and *cev1* (asterisks). (**D)** Box plot summary of primary root diameter at the onset of differentiation (*n* = 31). **(E)** Lignin deposition visualized by phloroglucinol stain (fuchsia) in primary roots of indicated genotypes. Letters in (B, D) denote statistically significant differences among samples determined by ANOVA followed by Tukey’s HSD test. Scale bar:100 μm (E).

**Additional file 9: Table S4 A-F**. Statistical analysis of root vascular penetrations upon Fo5176 pSIX1::GFP infection. Repeated measures two-way ANOVA with post-hoc Tukey test for multiple comparisons corresponding to root vascular penetration events *(p-value* < 0.05 *, 0.01 **, 0.001 ***, 0.0001 ****). Days on which there were no statistically significant differences (*p-value > 0.05)* are not included in the tables*.*

**Additional file 10: Table S5**. Differentially expressed genes in *ctl1-2* mutant compared to WT. (Sheet 1) Significant DEGs in *ctl1-2* 14 day-old roots compared to its WT (Col-0). Logarithmic fold-change (logFC) and corrected *p*-value (FDR) are presented. Genes encoding proteins containing a CAZY-domain, related with plant cell wall, or with hormone biology are highlighted in yellow, orange and green, respectively. (Sheet 2) GO-enrichment of all upregulated genes from (Sheet1) GO-ID: Gene ontology identifier, GO-term: Gene ontology descriptive term; % of associated genes: percentage of genes in that GO-term that are present in the analyzed cluster; No. of genes: absolute number of genes associated to the GO-term.

**Additional file 11: Figure S6**. Fo5176 infection does not induce callose deposition in roots. Representative images of callose deposition in roots in response to Fo5176 colonization. At 7dpt, Arabidopsis roots were stained with aniline blue to visualize callose deposits (arrow heads). Scale bar = 200 μm. The experiment was performed three times with similar results.

**Additional file 12: Table S6 A-B**. Statistical analysis of disease scoring symptoms upon *Ralstonia solanacearum* GMI1000 infection. Fisher’s exact contingency tests comparing different disease scoring categories corresponding to Figure 7 at 22 dpi (p-value < 0.05 *, 0.01 **, 0.001 ***, 0.0001 ****).

**Additional file 13: Table S7**. Comparison of published Arabidopsis root-Fo5176 transcriptomic studies. All published Arabidopsis root DEGs in response to Fo5176 (Chen et al. (2dpt) [12] and Lyons et al. (1dpt and 6dpt) [13]) were compared to the DEGs we obtained in our study. Genes highlighted in grey are identified as DEGs in our transcriptomic. Cell wall- and hormone-related DEGs reported in any of the previous studies and ours are highlighted in orange and green, respectively.

**Additional file 14: Table S8.** Primers used in this study

## Data Availability

The datasets generated during the current study are available in the GEO repository (GSE168919; https://www.ncbi.nlm.nih.gov/geo/query/acc.cgi?acc=GSE168919) [104]

## Abbreviations

CW: Cell wall
Fo: *Fusarium oxysporum*
SA: Salicylic acid
JA: Jasmonic acid
ET: Ethylene
ROS: Reactive oxygen species
CESA: Cellulose synthase subunit
CTL1: Chitinase-like1
GPI: Glycophosphatidyl-inositol
COB: Cobra
KOR1: Korrigan1
