# Supplementary figures and images for: A primary cell wall cellulose-dependent defense mechanism against vascular pathogens revealed by time-resolved dual transcriptomics
Source: BMC Biol. 2021 Aug 17;19:161. doi: 10.1186/s12915-021-01100-6 (PMC8371875; doi:10.1186/s12915-021-01100-6)

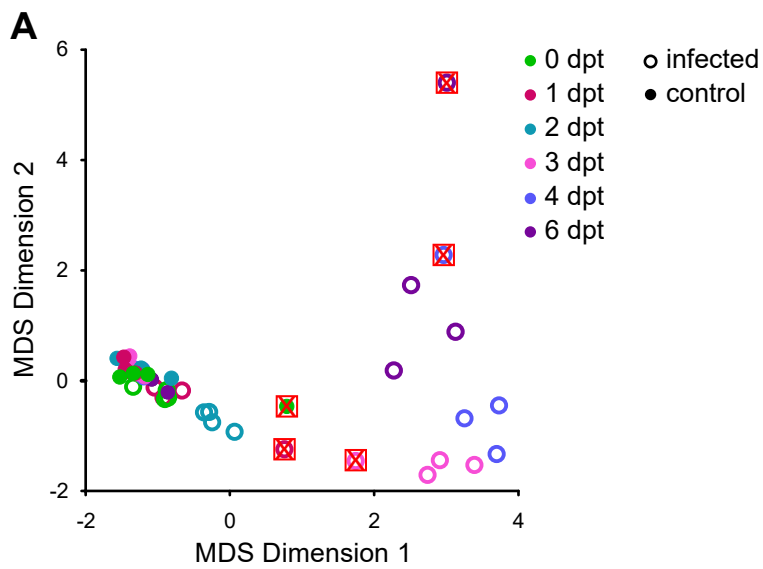

Supplement: Supplementary file 2 — Additional file 2: Figure S1. Complete MDS-Analysis of Arabidopsis transcriptional profiles. Multidimensional scaling analysis (MDS) of transcriptional profiles of Arabidopsis. Samples clearly deviating from the rest of the samples at the same time point and condition (surrounded by a red square) were not used for further analysis. [file 12915_2021_1100_MOESM2_ESM.pdf]

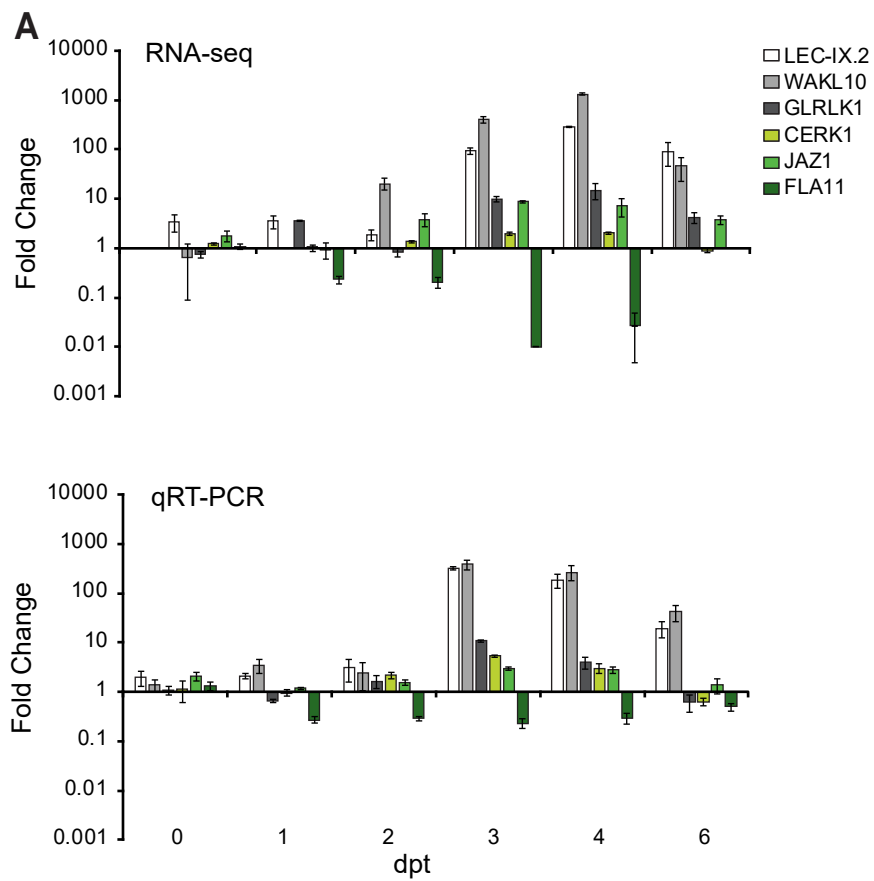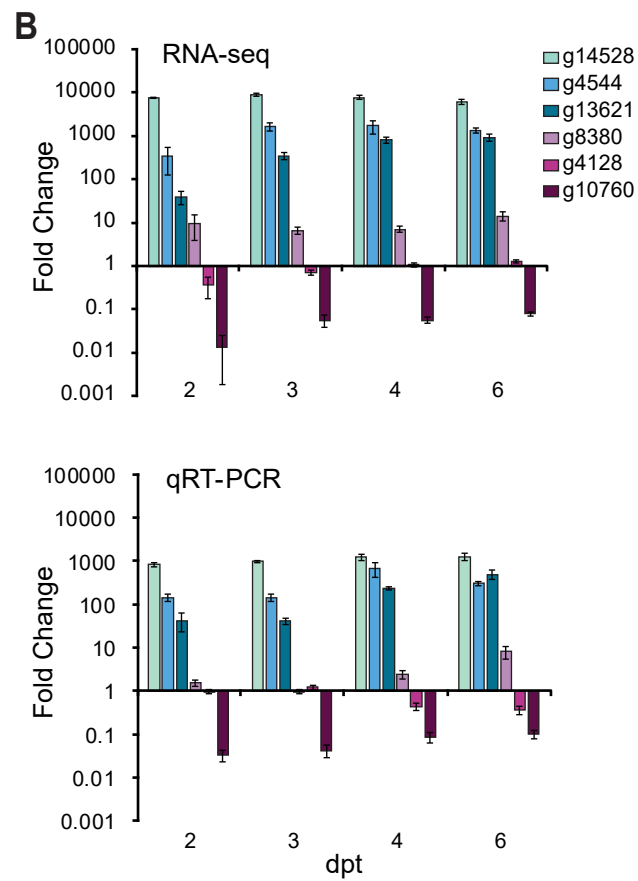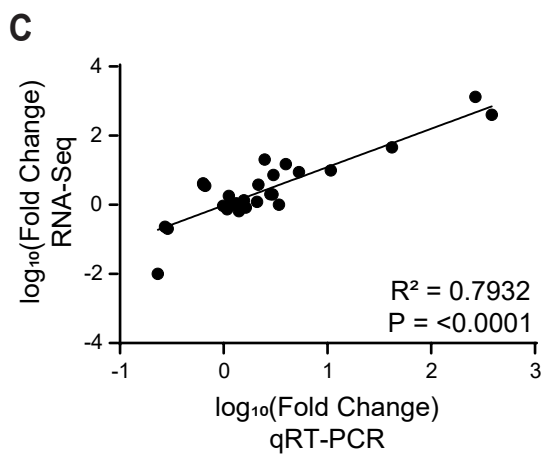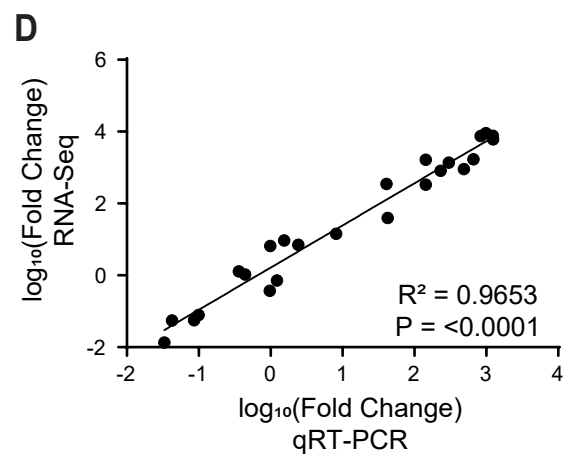

Supplement: Supplementary file 5 — Additional file 5: Figure S2. RNAseq validation by qRT-PCR. (A) and (B) The expression of 6 randomly picked DEGs from Arabidopsis (A) or Fo5176 (B) expressed at different levels based on the RNA-sequencing (left panels) was confirmed by qRT-PCR (right panels) using de novo generated RNA samples. The qRT-PCR-based expression of each gene was determined relative to the corresponding reference gene; i.e. At GAPDH in (A) and Fo5176 β-Tub in (B). (C) and (D) Correlation (log10 fold changes) between RNAseq and qPCR derived expression data from Arabidopsis (A and C) and Fo5176 (B and D). Very good Pearson correlations of r = 0.79 for Arabidopsis and r = 0.97 for the fungus were obtained (p-value < 0.0001 in both cases). Linear trend lines are depicted in black. [file 12915_2021_1100_MOESM5_ESM.pdf]

**A**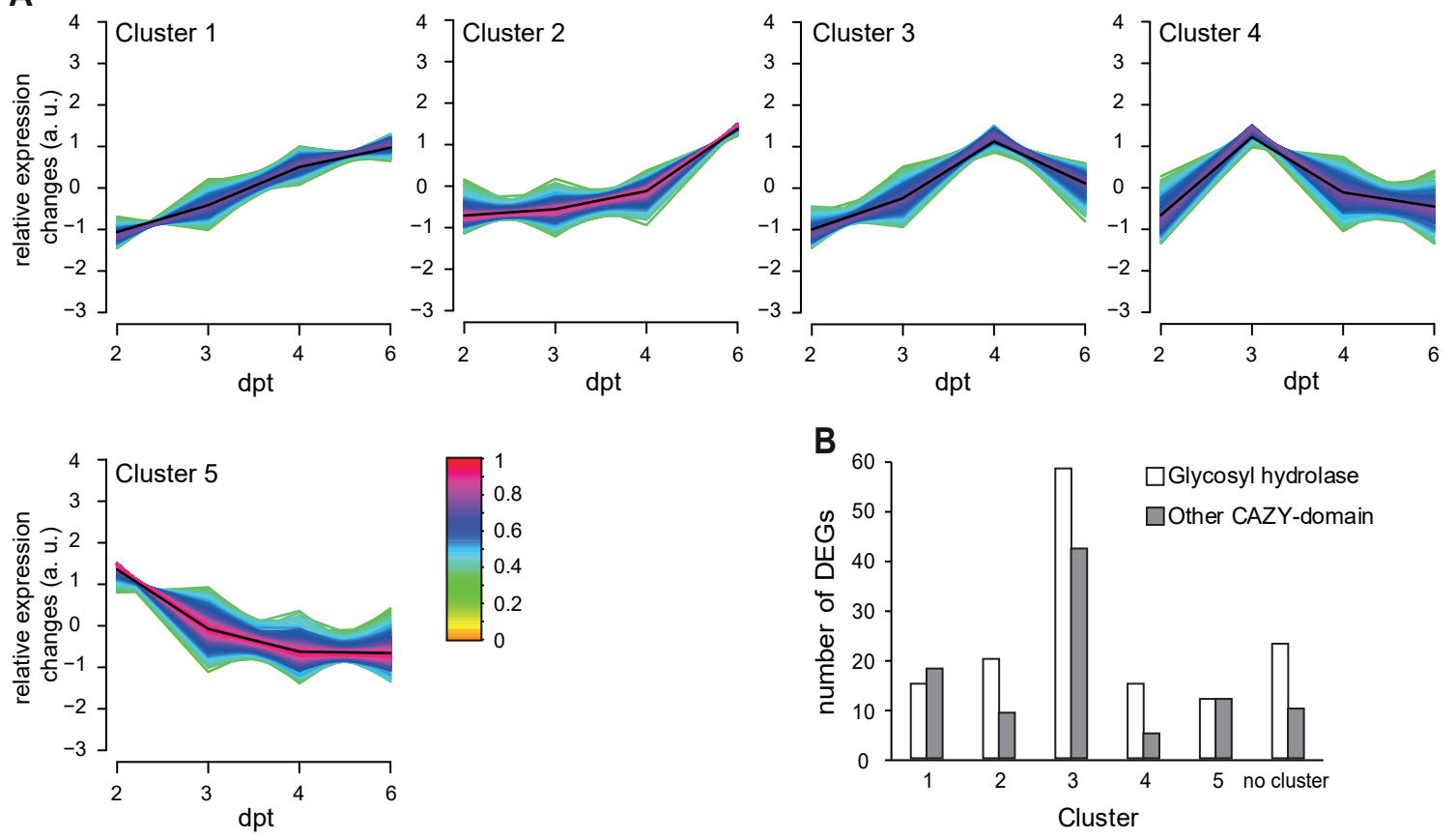**B**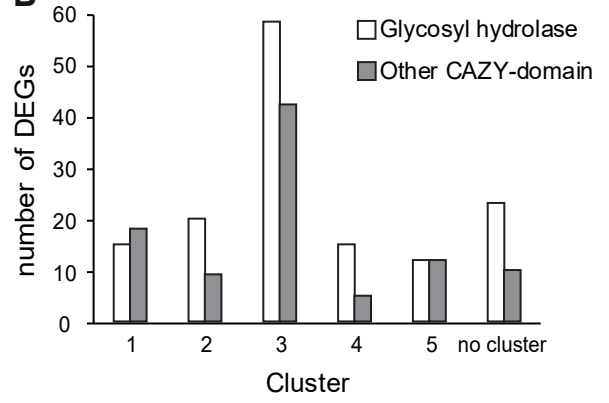

Supplement: Supplementary file 6 — Additional file 6: Figure S3. Temporal dynamics of Fo5176 DEGs during root infection reveal a significant alteration of proteins containing catalytic and carbohydrate-binding modules (CAZY). (A) Clusters of Fo5176 coexpressed DEGs during infection using fuzzy c-means clustering. (B) Number of DEGs encoding proteins with carbohydrate active enzyme-domains (CAZY) and glycosyl hydrolases in the different clusters. DEGs with the entry “no cluster” were not associated to a cluster by the clustering algorithm. [file 12915_2021_1100_MOESM6_ESM.pdf]

Supplemental Figure 4

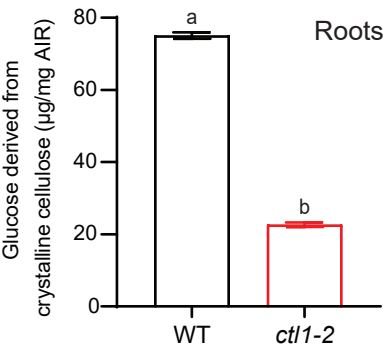

Supplement: Supplementary file 7 — Additional file 7: Figure S4. ctl1-2 is impaired in crystalline cellulose deposition in the root. Cellulose content of roots from 10 day-old light-grown plants represented as ug of D-glucose derived from crystalline cellulose per mg of dried alcohol-insoluble residue (AIR) [102]. N = 2 biological replicates +/- standard deviation; 3 technical replicates per biological replicate. Welch’s unpaired t-test; ***P-value = 0.0004. [file 12915_2021_1100_MOESM7_ESM.pdf]

Supplemental Figure 5

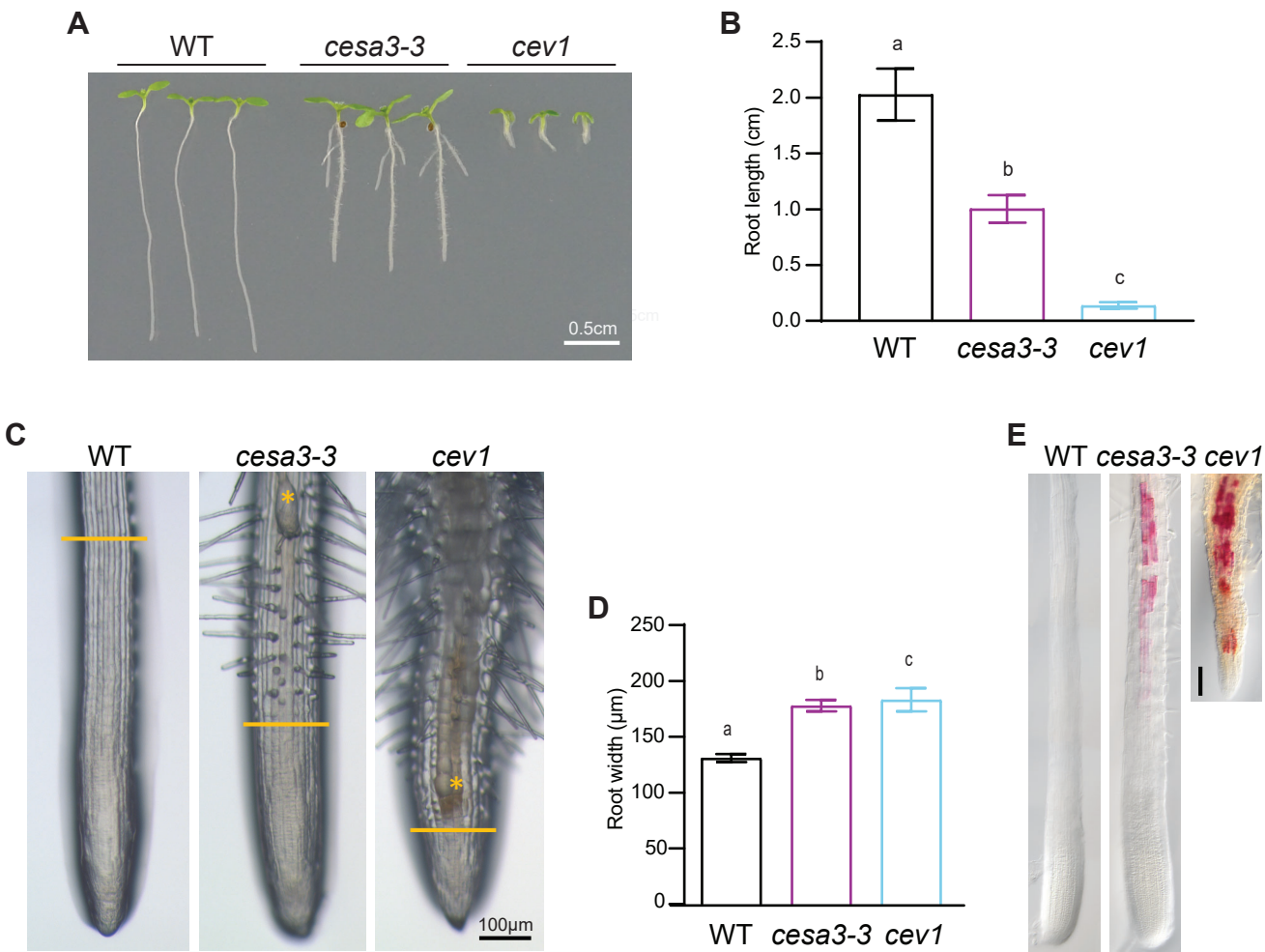

Supplement: Supplementary file 8 — Additional file 8: Figure S5. Characterization of cesa3-3. (A) Representative images of 7 day-old WT, cesa3-3 and cev1 seedlings. (B) Primary root length box plots of indicated genotypes. Medians are represented inside the boxes by solid lines, and circles depict individual measurements (n = 38-61). (C) Representative primary root images specifying the initiation of the differentiation zone by the appearance of root hairs (dashed line), and bulging cells in cesa3-3 and cev1 (asterisks). (D) Box plot summary of primary root diameter at the onset of differentiation (n = 31). (E) Lignin deposition visualized by phloroglucinol stain (fuchsia) in primary roots of indicated genotypes. Letters in (B, D) denote statistically significant differences among samples determined by ANOVA followed by Tukey’s HSD test. Scale bar:100 μm (E). [file 12915_2021_1100_MOESM8_ESM.pdf]

Supplemental Figure 6

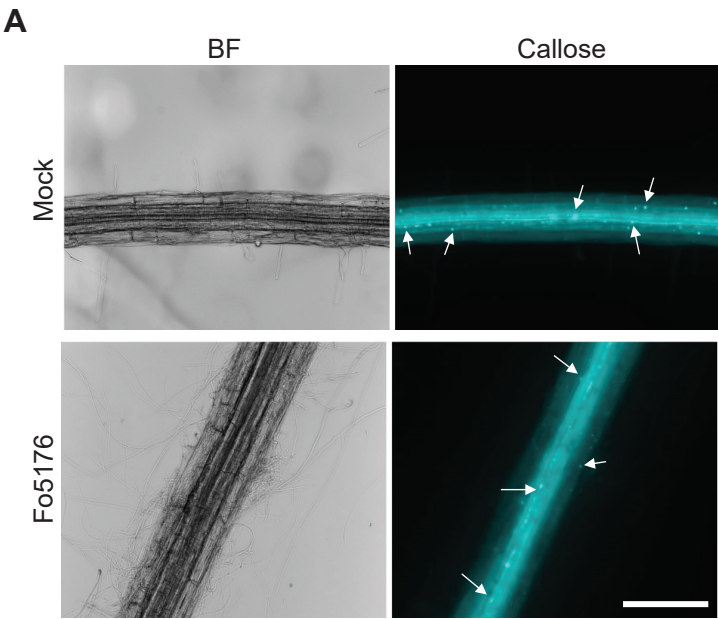

Supplement: Supplementary file 11 — Additional file 11: Figure S6. Fo5176 infection does not induce callose deposition in roots. Representative images of callose deposition in roots in response to Fo5176 colonization. At 7dpt, Arabidopsis roots were stained with aniline blue to visualize callose deposits (arrow heads). Scale bar = 200 μm. The experiment was performed three times with similar results. [file 12915_2021_1100_MOESM11_ESM.pdf]
