# Supplementary material for: A primary cell wall cellulose-dependent defense mechanism against vascular pathogens revealed by time-resolved dual transcriptomics
Source: BMC Biol. 2021 Aug 17;19:161. doi: 10.1186/s12915-021-01100-6 (PMC8371875; doi:10.1186/s12915-021-01100-6)
Supplement: Supplementary file 9 — Additional file 9: Table S4 A-F. Statistical analysis of root vascular penetrations upon Fo5176 pSIX1::GFP infection. Repeated measures two-way ANOVA with post-hoc Tukey test for multiple comparisons corresponding to root vascular penetration events (p-value < 0.05 *, 0.01 **, 0.001 ***, 0.0001 ****). Days on which there were no statistically significant differences (p-value > 0.05) are not included in the tables. [file 12915_2021_1100_MOESM9_ESM.docx]

**Table S4. Statistical analysis of root vascular penetrations upon Fo5176 pSIX1::GFP infection.**

**Table S4A (linked to Figure 3A).**

| **Two-way RM ANOVA (Tukey test)** | **Adjusted p-value** |
| --- | --- |
| **4 dpt** | |
| **WT vs. *ctl1-2*** | **ns** |
| **WT vs. *cobra-6*** | ***** |
| **WT vs. *prc1-1*** | ***** |
| ***ctl1-2* vs. *cobra-6*** | **ns** |
| ***ctl1-2* vs. *prc1-1*** | **ns** |
| ***cobra-6* vs. *prc1-1*** | **ns** |
| **5 dpt** | |
| **WT vs. *ctl1-2*** | ******** |
| **WT vs. *cobra-6*** | ******** |
| **WT vs. *prc1-1*** | ******* |
| ***ctl1-2* vs. *cobra-6*** | **ns** |
| ***ctl1-2* vs. *prc1-1*** | **ns** |
| ***cobra-6* vs. *prc1-1*** | **ns** |
| **6 dpt** | |
| **WT vs. *ctl1-2*** | ******** |
| **WT vs. *cobra-6*** | ******** |
| **WT vs. *prc1-1*** | ******** |
| ***ctl1-2* vs. *cobra-6*** | **ns** |
| ***ctl1-2* vs. *prc1-1*** | **ns** |
| ***cobra-6* vs. *prc1-1*** | **ns** |
| **7 dpt** | |
| **WT vs. *ctl1-2*** | ********* |
| **WT vs. *cobra-6*** | ******** |
| ***WT* vs. *prc1-1*** | ******** |
| ***ctl1-2* vs. *cobra-6*** | **ns** |
| ***ctl1-2* vs. *prc1-1*** | **ns** |
| ***cobra-6* vs. *prc1-1*** | **ns** |

**Table S4B (linked to Figure 3B).**

| **Two-way RM ANOVA (Tukey test)** | **Adjusted p-value** |
| --- | --- |
| **4 dpt** | |
| **WT JGP vs. *cesa3-3*** | **ns** |
| **WT JGP vs. *kor1-4*** | ****** |
| ***cesa3-3* vs. *kor1-4*** | **ns** |
| **5 dpt** | |
| **WT JGP vs. *cesa3-3*** | ******** |
| **WT JGP vs. *kor1-4*** | ******** |
| ***cesa3-3* vs. *kor1-4*** | **ns** |
| **6 dpt** | |
| **WT JGP vs. *cesa3-3*** | ******** |
| **WT JGP vs. *kor1-4*** | ******** |
| ***cesa3-3* vs. *kor1-4*** | **ns** |
| **7 dpt** | |
| **WT JGP vs. *cesa3-3*** | ******** |
| **WT JGP vs. *kor1-4*** | ******** |
| ***cesa3-3* vs. *kor1-4*** | **ns** |

**Table S4C (linked to Figure 4C).**

| **Two-way RM ANOVA (Tukey test)** | **Adjusted p-value** |
| --- | --- |
| **4 dpt** | |
| **WT vs. *4cl1-1*** | **ns** |
| **WT vs. *4cl1-2*** | **ns** |
| **WT vs. *4cl2-1*** | **ns** |
| **WT vs. *ccoAomt1-5*** | **ns** |
| **WT vs. *c4h3-1*** | ***** |
| ***4cl1-1* vs. *4cl1-2*** | **ns** |
| ***4cl1-1* vs. *4cl2-1*** | **ns** |
| ***4cl1-1* vs. *ccoAomt1-5*** | **ns** |
| ***4cl1-1* vs. *c4h3-1*** | ****** |
| ***4cl1-2* vs. *4cl2-1*** | **ns** |
| ***4cl1-2* vs. *ccoAomt1-5*** | **ns** |
| ***4cl1-2* vs. *c4h3-1*** | **ns** |
| ***4cl2-1* vs. *ccoAomt1-5*** | **ns** |
| ***4cl2-1* vs. *c4h3-1*** | **ns** |
| ***ccoAomt1-5* vs. *c4h3-1*** | ****** |
| **5 dpt** | |
| **WT vs. *4cl1-1*** | **ns** |
| **WT vs. *4cl1-2*** | **ns** |
| **WT vs. *4cl2-1*** | **ns** |
| **WT vs. *ccoAomt1-5*** | ***** |
| **WT vs. *c4h3-1*** | ******** |
| ***4cl1-1* vs. *4cl1-2*** | **ns** |
| ***4cl1-1* vs. *4cl2-1*** | ***** |
| ***4cl1-1* vs. *ccoAomt1-5*** | ***** |
| ***4cl1-1* vs. *c4h3-1*** | ******** |
| ***4cl1-2* vs. *4cl2-1*** | **ns** |
| ***4cl1-2* vs. *ccoAomt1-5*** | **ns** |
| ***4cl1-2* vs. *c4h3-1*** | ****** |
| ***4cl2-1* vs. *ccoAomt1-5*** | **ns** |
| ***4cl2-1* vs. *c4h3-1*** | ****** |
| ***ccoAomt1-5* vs. *c4h3-1*** | ***** |
| **6 dpt** | |
| **WT vs. *4cl1-1*** | **ns** |
| **WT vs. *4cl1-2*** | **ns** |
| **WT vs. *4cl2-1*** | ***** |
| **WT vs. *ccoAomt1-5*** | ****** |
| **WT vs. *c4h3-1*** | ******** |
| ***4cl1-1* vs. *4cl1-2*** | **ns** |
| ***4cl1-1* vs. *4cl2-1*** | **ns** |
| ***4cl1-1* vs. *ccoAomt1-5*** | **ns** |
| ***4cl1-1* vs. *c4h3-1*** | ******** |
| ***4cl1-2* vs. *4cl2-1*** | **ns** |
| ***4cl1-2* vs. *ccoAomt1-5*** | **ns** |
| ***4cl1-2* vs. *c4h3-1*** | ******** |
| ***4cl2-1* vs. *ccoAomt1-5*** | **ns** |
| ***4cl2-1* vs. *c4h3-1*** | ******** |
| ***ccoAomt1-5* vs. *c4h3-1*** | ******** |
| **7 dpt** | |
| **WT vs. *4cl1-1*** | **ns** |
| **WT vs. *4cl1-2*** | **ns** |
| **WT vs. *4cl2-1*** | **ns** |
| **WT vs. *ccoAomt1-5*** | **ns** |
| **WT vs. *c4h3-1*** | ******** |
| ***4cl1-1* vs. *4cl1-2*** | **ns** |
| ***4cl1-1* vs. *4cl2-1*** | **ns** |
| ***4cl1-1* vs. *ccoAomt1-5*** | **ns** |
| ***4cl1-1* vs. *c4h3-1*** | ******** |
| ***4cl1-2* vs. *4cl2-1*** | **ns** |
| ***4cl1-2* vs. *ccoAomt1-5*** | **ns** |
| ***4cl1-2* vs. *c4h3-1*** | ******** |
| ***4cl2-1* vs. *ccoAomt1-5*** | **ns** |
| ***4cl2-1* vs. *c4h3-1*** | ******** |
| ***ccoAomt1-5* vs. *c4h3-1*** | ******** |

**Table S4D (linked to Figure 5A).**

| **Two-way RM ANOVA (Tukey test)** | **Adjusted p-value** |
| --- | --- |
| **5 dpt** | |
| **WT vs. *ctl1-2*** | ****** |
| **WT vs. *aos*** | **ns** |
| **WT vs. *ctl1-2 aos*** | **ns** |
| ***ctl1-2* vs. *aos*** | ******* |
| ***ctl1-2* vs. *ctl1-2 aos*** | **ns** |
| ***aos* vs. *ctl1-2 aos*** | **ns** |
| **6 dpt** | |
| **WT vs. *ctl1-2*** | ******** |
| **WT vs. *aos*** | **ns** |
| **WT vs. *ctl1-2 aos*** | ******** |
| ***ctl1-2* vs. *aos*** | ******** |
| ***ctl1-2* vs. *ctl1-2 aos*** | **ns** |
| ***aos* vs. *ctl1-2 aos*** | ******* |
| **7 dpt** | |
| **WT vs. *ctl1-2*** | ******** |
| **WT vs. *aos*** | **ns** |
| **WT vs. *ctl1-2 aos*** | ******** |
| ***ctl1-2* vs. *aos*** | ******** |
| ***ctl1-2* vs. *ctl1-2 aos*** | **ns** |
| ***aos* vs. *ctl1-2 aos*** | ******** |

**Table S4E (linked to Figure 5B).**

| **Two-way RM ANOVA (Tukey test)** | **Adjusted p-value** |
| --- | --- |
| **5 dpt** | |
| **WT vs. *ctl1-2*** | ******* |
| **WT vs. *coi1-34*** | ***** |
| **WT vs. *ctl1-2 coi1-34*** | ******** |
| ***ctl1-2* vs. *coi1-34*** | **ns** |
| ***ctl1-2* vs. *ctl1-2 coi1-34*** | **ns** |
| ***coi1-34* vs. *ctl1-2 coi1-34*** | **ns** |
| **6 dpt** | |
| **WT vs. *ctl1-2*** | ******** |
| **WT vs. *coi1-34*** | ****** |
| **WT vs. *ctl1-2 coi1-34*** | ******** |
| ***ctl1-2* vs. *coi1-34*** | ****** |
| ***ctl1-2* vs. *ctl1-2 coi1-34*** | **ns** |
| ***coi1-34* vs. *ctl1-2 coi1-34*** | ******** |
| **7 dpt** | |
| **WT vs. *ctl1-2*** | ******** |
| **WT vs. *coi1-34*** | ***** |
| **WT vs. *ctl1-2 coi1-34*** | ******** |
| ***ctl1-2* vs. *coi1-34*** | ******** |
| ***ctl1-2* vs. *ctl1-2 coi1-34*** | **ns** |
| ***coi1-34* vs. *ctl1-2 coi1-34*** | ******** |

**Table S4F (linked to Figure 6A).**

| **Two-way RM ANOVA (Tukey test)** | **Adjusted p-value** |
| --- | --- |
| **4 dpt** | |
| **WT vs. *ctl1-2*** | ***** |
| **WT vs. *ein2-5*** | **ns** |
| **WT vs. *ctl1-2 ein2-5*** | **ns** |
| ***ctl1-2* vs. *ein2-5*** | ******** |
| ***ctl1-2* vs. *ctl1-2 ein2-5*** | ******** |
| ***ein2-5* vs. *ctl1-2 ein2-5*** | **ns** |
| **5 dpt** | |
| **WT vs. *ctl1-2*** | ******** |
| **WT vs. *ein2-5*** | ******* |
| **WT vs. *ctl1-2 ein2-5*** | **ns** |
| ***ctl1-2* vs. *ein2-5*** | ******** |
| ***ctl1-2* vs. *ctl1-2 ein2-5*** | ******** |
| ***ein2-5* vs. *ctl1-2 ein2-5*** | ******* |
| **6 dpt** | |
| **WT vs. *ctl1-2*** | ******** |
| **WT vs. *ein2-5*** | ******** |
| **WT vs. *ctl1-2 ein2-5*** | **ns** |
| ***ctl1-2* vs. *ein2-5*** | ******** |
| ***ctl1-2* vs. *ctl1-2 ein2-5*** | ******** |
| ***ein2-5* vs. *ctl1-2 ein2-5*** | ******** |
| **7 dpt** | |
| **WT vs. *ctl1-2*** | ******** |
| **WT vs. *ein2-5*** | ******** |
| **WT vs. *ctl1-2 ein2-5*** | **ns** |
| ***ctl1-2* vs. *ein2-5*** | ******** |
| ***ctl1-2* vs. *ctl1-2 ein2-5*** | ******** |
| ***ein2-5* vs. *ctl1-2 ein2-5*** | ******** |
