# Supplementary material for: A primary cell wall cellulose-dependent defense mechanism against vascular pathogens revealed by time-resolved dual transcriptomics
Source: BMC Biol. 2021 Aug 17;19:161. doi: 10.1186/s12915-021-01100-6 (PMC8371875; doi:10.1186/s12915-021-01100-6)
Supplement: Supplementary file 12 — Additional file 12: Table S6 A-B. Statistical analysis of disease scoring symptoms upon Ralstonia solanacearum GMI1000 infection. Fisher’s exact contingency tests comparing different disease scoring categories corresponding to Figure 7 at 22 dpi (p-value < 0.05 *, 0.01 **, 0.001 ***, 0.0001 ****). [file 12915_2021_1100_MOESM12_ESM.docx]

**Table S6. Statistical analysis of disease scoring symptoms upon *Ralstonia solanacearum* GMI1000 infection**

**Table S6A (linked to Figure 7A).**

| **Fisher’s exact contingency test** | **Adjusted p-value** | |
| --- | --- | --- |
|  | **≤1 vs. >3 ≤4** | **>1 ≤2 vs. >2 ≤3** |
| **WT vs. *ctl1-2*** | ******** | **ns** |
| **WT vs *cobra-6*** | ******** | **ns** |
| **WT vs. *prc1-1*** | ******** | **ns** |
| ***ctl1-2* vs. *cobra-6*** | **ns** | **ns** |
| ***ctl1-2* vs. *prc1-1*** | ******** | **ns** |
| ***cobra-6* vs *prc1-1*** | ******** | **ns** |

**Table S6B (linked to Figure 7B).**

| **Fisher’s exact contingency test** | **Adjusted p-value** | |
| --- | --- | --- |
|  | **≤1 vs. >3 ≤4** | **>1 ≤2 vs. >2 ≤3** |
| **WT vs. *ctl1-2*** | ******** | **ns** |
| **WT vs *ein2-5*** | ******** | **ns** |
| **WT vs. *ctl1-2 ein2-5*** | ******** | **ns** |
| ***ctl1-2* vs. *ein2-5*** | **ns** | **ns** |
| ***ctl1-2* vs. *ctl1-2 ein2-5*** | **ns** | **ns** |
| ***ein2-5* vs *ctl1-2 ein2-5*** | **ns** | **ns** |
