# Supplementary material for: A primary cell wall cellulose-dependent defense mechanism against vascular pathogens revealed by time-resolved dual transcriptomics
Source: BMC Biol. 2021 Aug 17;19:161. doi: 10.1186/s12915-021-01100-6 (PMC8371875; doi:10.1186/s12915-021-01100-6)
Supplement: Supplementary file 14 — Additional file 14: Table S8. Primers used in this study [file 12915_2021_1100_MOESM14_ESM.docx]

**Table S8.** Primers used in this study

| **Gene name** | **Primer 1** | **Primer 2** |
| --- | --- | --- |
| *cesa3-3_Hpy188I (AT5G05170)^a^* | TGTTTGCCTATACTGTTTCCCA | GTGCACGAGGCTCTATGCTA |
| *coi1-34 (AT2G39940)^b^* | GGTTCTCTTTAGTCTTTAC | CAGACAACTATTTCGTTACC |
| *LEC-IX.2 (AT5G65600) qRT-PCR* | CCTCGCTAAGGTTCTGCCAT | CAAACCCGGAAGCCGAAATG |
| *WAKL10 (AT1G79680) qRT-PCR* | TCGTGCTAGCGGAGCTAATC | TCCTTGCAATTTTTGCGGCT |
| *GLRLK1 (AT4G21390) qRT-PCR* | CGCAGCCGAATCTTGAACAC | GAATGCTTGCAGGCTTGGTA |
| *CERK1 (AT3G21630) qRT-PCR* | GATTTCTGCGAAAGGTGCGG | CGGGTAACTATCACCGAGCC |
| *FLA11 (AT5G03170) qRT-PCR* | CTGAGAAAGGCGGCTCTGTT | ATGGCTGCAACGGTAGTGAT |
| *JAZ1 (AT1G19180) qRT-PCR* | TTCTGAGTTCGTCGGTAGCC | AGGCTTGCATGCCATTCCTA |
| *Fo5197.g14528 qRT-PCR* | GCCATTTCCAACCACGCTTT | TCATGGGGATGACGGTCGTA |
| *Fo5176.g4544 qRT-PCR* | GCTGGCACTTACACTCCCTT | TGCTCTCATCACAGGCGAAG |
| *Fo5176.g13621 qRT-PCR* | CCCACCCCTCTTACTCTCCA | GTGCGAGATACACCGAGGAG |
| *Fo5176.g8380 qRT-PCR* | AAGGCTATGGCCAAGTTCCC | CAAGTCCGACATGAGTCCCC |
| *Fo5176.g4128 qRT-PCR* | CATGAGTTGCCGTTCTCGTG | CGCCGAGGTTGGTAACAGTA |
| *Fo5176.g10760 qRT-PCR* | TCGACCGAGACTGTCTTTGC | CAGCCCTTGACGACATGTGA |
| *Fo5176.g4360 qRT-PCR* | AACTCCGATGAGACCTTCTG | GACATGACAGCAGAAACGAG |
| *AT_GAPDH_3’  qRT-PCR* | TTGGTGACAACAGGTCAAGCA | AAACTTGTCGCTCAATGCAATC |
| *AT_GAPDH_5’  qRT-PCR* | TCTCGATCTCAATTTCGCAAAA | CGAAACCGTTGATTCCGATTC |

^a^ Amplification products were digested with *Hpy188I*; cut products of 212, 58, 48bp were confirmed as WT, while cut products of 261 and 58bp were confirmed as *cesa3-3* mutants.

^b^ These primers were used for gene-specific sequencing to confirm point-mutation
